# Conditional Rényi Divergences and Horse Betting

**DOI:** 10.3390/e22030316

**Published:** 2020-03-11

**Authors:** Cédric Bleuler, Amos Lapidoth, Christoph Pfister

**Affiliations:** Signal and Information Processing Laboratory, ETH Zurich, 8092 Zurich, Switzerland; cedric.bleuler@gmail.com (C.B.); lapidoth@isi.ee.ethz.ch (A.L.)

**Keywords:** conditional Rényi divergence, horse betting, Kelly gambling, Rényi divergence, Rényi mutual information

## Abstract

Motivated by a horse betting problem, a new conditional Rényi divergence is introduced. It is compared with the conditional Rényi divergences that appear in the definitions of the dependence measures by Csiszár and Sibson, and the properties of all three are studied with emphasis on their behavior under data processing. In the same way that Csiszár’s and Sibson’s conditional divergence lead to the respective dependence measures, so does the new conditional divergence lead to the Lapidoth–Pfister mutual information. Moreover, the new conditional divergence is also related to the Arimoto–Rényi conditional entropy and to Arimoto’s measure of dependence. In the second part of the paper, the horse betting problem is analyzed where, instead of Kelly’s expected log-wealth criterion, a more general family of power-mean utility functions is considered. The key role in the analysis is played by the Rényi divergence, and in the setting where the gambler has access to side information, the new conditional Rényi divergence is key. The setting with side information also provides another operational meaning to the Lapidoth–Pfister mutual information. Finally, a universal strategy for independent and identically distributed races is presented that—without knowing the winning probabilities or the parameter of the utility function—asymptotically maximizes the gambler’s utility function.

## 1. Introduction

As shown by Kelly [1,2], many of Shannon’s information measures appear naturally in the context of horse gambling when the gambler’s utility function is expected log-wealth. Here, we show that under a more general family of utility functions, gambling also provides a context for some of Rényi’s information measures. Moreover, the setting where the gambler has side information motivates a new Rényi-like conditional divergence, which we study and compare to other conditional divergences. The proposed family of utility functions in the context of gambling with side information also provides another operational meaning to the Rényi-like mutual information that was recently proposed by Lapidoth and Pfister [3]: it measures the gambler’s gain from the side information as measured by the increase in the minimax value of the two-player zero-sum game in which the bookmaker picks the odds and the gambler then places the bets based on these odds and her side information.

Deferring the gambling-based motivation to the second part of the paper, we first describe the different conditional divergences and study some of their properties with emphasis on their behavior under data processing. We also show that the new conditional Rényi divergence relates to the Lapidoth–Pfister mutual information in much the same way that Csiszár’s and Sibson’s conditional divergences relate to their corresponding mutual informations. Before discussing the conditional divergences, we first recall other information measures.

The Kullback–Leibler divergence (or relative entropy) is an important concept in information theory and statistics [2,4,5,6]. It is defined between two probability mass functions (PMFs) *P* and *Q* over a finite set X as

(1) $$\begin{array}{c} D\left(P\|Q\right)≜\sum_{x\in X}P\left(x\right)\log\frac{P(x)}{Q(x)}, \end{array}$$

where log(·) denotes the base-2 logarithm. Defining a conditional Kullback–Leibler divergence is straightforward because, as simple algebra shows, the two natural approaches lead to the same result:

(2) $$\begin{array}{cc} D(P_{Y|X}\|Q_{Y|X}|P_{X}) & ≜\sum_{x\in \mathrm{supp}(P_{X})}P\left(x\right)\;D\left(P_{Y|X=x}\|Q_{Y|X=x}\right) \end{array}$$

(3) $$\begin{array}{cc} \; & =D(P_{X}P_{Y|X}\|P_{X}Q_{Y|X}), \end{array}$$

where supp(P)≜{x∈X:P(x)>0} denotes the support of *P*, and in (3) and throughout $P_{X}P_{Y|X}$ denotes the PMF on X×Y that assigns (x,y) the probability $P_{X}\left(x\right)\;P_{Y|X}\left(y|x\right)$.

The Rényi divergence of order α [7,8] between two PMFs *P* and *Q* is defined for all positive α’s other than one as

(4) $$\begin{array}{c} D_{\alpha }\left(P\|Q\right)≜\frac{1}{\alpha -1}\log\sum_{x\in X}P{\left(x\right)}^{\alpha }\;Q{\left(x\right)}^{1-\alpha }. \end{array}$$

A conditional Rényi divergence can be defined in more than one way. In this paper, we consider the following three definitions, two classic and one new:

(5) $$\begin{array}{cc} D_{\alpha }^{c}(P_{Y|X}\|Q_{Y|X}\left|P_{X}\right) & ≜\sum_{x\in \mathrm{supp}(P_{X})}P\left(x\right)\;D_{\alpha }\left(P_{Y|X=x}\|Q_{Y|X=x}\right), \end{array}$$

(6) $$\begin{array}{cc} D_{\alpha }^{s}(P_{Y|X}\|Q_{Y|X}\left|P_{X}\right) & ≜D_{\alpha }\left(P_{X}P_{Y|X}\|P_{X}Q_{Y|X}\right), \end{array}$$

(7) $$\begin{array}{cc} D_{\alpha }^{l}(P_{Y|X}\|Q_{Y|X}\left|P_{X}\right) & ≜\frac{\alpha }{\alpha -1}\log\sum_{x\in \mathrm{supp}(P_{X})}P\left(x\right)\;2^{\frac{\alpha -1}{\alpha }D_{\alpha }\left(P_{Y|X=x}\|Q_{Y|X=x}\right)}, \end{array}$$

where (5) is inspired by Csiszár [9]; (6) is inspired by Sibson [10]; and (7) is motivated by the horse betting problem discussed in Section 9. The first two conditional Rényi divergences were used to define the Rényi measures of dependence of Csiszár $I_{\alpha }^{c}\left(X;Y\right)$[9] and of Sibson $I_{\alpha }^{s}\left(X;Y\right)$ [10]:

(8) $$\begin{array}{cc} I_{\alpha }^{c}\left(X;Y\right) & ≜\min_{Q_{Y}}D_{\alpha }^{c}(P_{Y|X}\|Q_{Y}\left|P_{X}\right), \end{array}$$

(9) $$\begin{array}{cc} I_{\alpha }^{s}\left(X;Y\right) & ≜\min_{Q_{Y}}D_{\alpha }^{s}(P_{Y|X}\|Q_{Y}\left|P_{X}\right), \end{array}$$

where the minimization is over all PMFs on the set Y. (Gallager’s $E_{0}$ function [11] and $I_{\alpha }^{s}\left(X;Y\right)$ are in one-to-one correspondence; see (65) below.) The analogous minimization of $D_{\alpha }^{l}\left(\cdot \right)$ leads to the Lapidoth–Pfister mutual information $J_{\alpha }\left(X;Y\right)$ [3]:

(10) $$\begin{array}{cc} J_{\alpha }\left(X;Y\right) & ≜\min_{Q_{X},\;Q_{Y}}D_{\alpha }\left(P_{XY}\|Q_{X}Q_{Y}\right) \end{array}$$

(11) $$\begin{array}{cc} \; & =\min_{Q_{Y}}D_{\alpha }^{l}(P_{Y|X}\|Q_{Y}\left|P_{X}\right), \end{array}$$

where (11) is proved in Proposition 5.

The first part of the paper is structured as follows: In Section 2, we discuss some preliminaries. In Section 3, Section 4 and Section 5, we study the properties of the three conditional Rényi divergences and their associated measure of dependence. In Section 6, we express the Arimoto–Rényi conditional entropy $H_{\alpha }\left(X|Y\right)$ and the Arimoto measure of dependence $I_{\alpha }^{a}\left(X;Y\right)$[12] in terms of $D_{\alpha }^{l}(P_{X|Y}\|U_{X}\left|P_{Y}\right)$. In Section 7, we relate the conditional Rényi divergences to each other and discuss the relations between the Rényi dependence measures.

The second part of the paper deals with horse gambling under our proposed family of power-mean utility functions. It is in this context that the Rényi divergence (Theorem 9) and the conditional Rényi divergence $D_{\alpha }^{l}\left(\cdot \right)$ (Theorem 10) appear naturally.

More specifically, consider a horse race with a finite nonempty set of horses X, where a bookmaker offers odds o(x)-for-1 on each horse x∈X, where o:X→(0,∞) [2] (Section 6.1). A gambler spends all her wealth placing bets on the horses. The fraction of her wealth that she bets on Horse x∈X is denoted b(x)≥0, which sums up to 1 over x∈X, and the PMF *b* is her “betting strategy.” The winning horse, which we denote *X*, is drawn according to the PMF *p*, where we assume p(x)>0 for all x∈X. The wealth relative (or end-to-beginning wealth ratio) is the random variable

(12) $$\begin{array}{c} S≜b(X)\;o(X). \end{array}$$

Hence, given an initial wealth γ, the gambler’s wealth after the race is γS. We seek betting strategies that maximize the utility function

(13) $$\begin{array}{c} U_{\beta }≜\left\{\begin{array}{cc} \frac{1}{\beta }\log E\left[S^{\beta }\right] & \mathrm{if}\;\beta \neq 0,\\ E[\log S] & \mathrm{if}\;\beta =0, \end{array}\right. \end{array}$$

where β∈R is a parameter that accounts for the risk sensitivity. This optimization generalizes the following cases:

(a) In the limit as β tends to −∞, we optimize the worst-case return. The optimal strategy is risk-free in the sense that *S* does not depend on the winning horse (see Proposition 8).
(b) If β=0, then we optimize E[logS], which is known as the doubling rate [2] (Section 6.1). The optimal strategy is proportional betting, i.e., to choose b=p (see Remark 4).
(c) If β=1, then we optimize E[S], the expected return. The optimal strategy is to put all the money on a horse that maximizes p(x)o(x) (see Proposition 9).
(d) In general, if β≥1, then it is optimal to put all the money on one horse (see Proposition 9). This is risky: if that horse loses, the gambler will go broke.
(e) In the limit as β tends to +∞, we optimize the best-case return. The optimal strategy is to put all the money on a horse that maximizes o(x) (see Proposition 10).

Note that, for β≠0 and η≜1−β, maximizing $U_{\beta }$ is equivalent to maximizing

(14) $$\begin{array}{c} E\left[\frac{S^{1-\eta }}{1-\eta }\right], \end{array}$$

which is known in the finance literature as Constant Relative Risk Aversion (CRRA) [13,14].

We refer to our utility function as “power mean” because it can be written as the logarithm of a weighted power mean [15,16]:

(15) $$\begin{array}{c} U_{\beta }=\log{\left[\sum_{x}p\left(x\right)\;{\left(b(x)\;o(x)\right)}^{\beta }\right]}^{\frac{1}{\beta }}. \end{array}$$

Because the power mean tends to the geometric mean as β tends to zero [15] (Problem 8.1), $U_{\beta }$ is continuous at β=0:

(16) $$\begin{array}{cc} \lim_{\beta \to 0}U_{\beta } & =\log\prod_{x}{\left(b(x)\;o(x)\right)}^{p(x)} \end{array}$$

(17) $$\begin{array}{cc} \; & =E[\log S]. \end{array}$$

Campbell [17,18] used an exponential cost function with a similar structure to (15) to provide an operational meaning to the Rényi entropy in source coding. Other information-theoretic applications of exponential moments were studied in [19].

The second part of the paper is structured as follows: In Section 8, we relate the utility function $U_{\beta }$ to the Rényi divergence (Theorem 9) and derive its optimal gambling strategy. In Section 9, we consider the situation where the gambler observes side information prior to betting, a situation that leads to the conditional Rényi divergence $D_{\alpha }^{l}\left(\cdot \right)$ (Theorem 10) and to a new operational meaning for the measure of dependence $J_{\alpha }\left(X;Y\right)$ (Theorem 11). In Section 10, we consider the situation where the gambler invests only part of her money. In Section 11, we present a universal strategy for independent and identically distributed (IID) races that requires neither knowledge of the winning probabilities nor of the parameter β of the utility function and yet asymptotically maximizes the utility function for all PMFs *p* and all β∈R.

## 2. Preliminaries

Throughout the paper, log(·) denotes the base-2 logarithm, X and Y are finite sets, $P_{XY}$ denotes a joint PMF over X×Y, $Q_{X}$ denotes a PMF over X, and $Q_{Y}$ denotes a PMF over Y. An expression of the form $P_{X}P_{Y|X}$ denotes the PMF on X×Y that assigns (x,y) the probability $P_{X}\left(x\right)\;P_{Y|X}\left(y|x\right)$. We use *P* and *Q* as generic PMFs over a finite set X. We denote by supp(P)≜{x∈X:P(x)>0} the support of *P*, and by P(X) the set of all PMFs over X. When clear from the context, we often omit sets and subscripts: for example, we write $\sum_{x}$ for $\sum_{x\in X}$, $\min_{Q_{X},\;Q_{Y}}$ for $\min_{\left(Q_{X},\;Q_{Y}\right)\in P\left(X\right)\times P\left(Y\right)}$, P(x) for $P_{X}\left(x\right)$, and P(y|x) for $P_{Y|X}\left(y|x\right)$. When P(x) is 0, we define the conditional probability P(y|x) as 1/|Y|. The conditional distribution of *Y* given X=x is denoted by $P_{Y|X=x}$, thus

(18) $$\begin{array}{cc} P_{Y|X=x}\left(y\right) & =P(y|x). \end{array}$$

We denote by 𝟙{condition} the indicator function that is one if the condition is satisfied and zero otherwise.

In the definition of the Kullback–Leibler divergence in (1), we use the conventions

(19) $$\begin{array}{c} 0\log\frac{0}{q}=0\;\forall \;q\geq 0,\;p\log\frac{p}{0}=\infty \;\forall \;p>0. \end{array}$$

In the definition of the Rényi divergence in (4), we read $P{\left(x\right)}^{\alpha }\;Q{\left(x\right)}^{1-\alpha }$ as $P{\left(x\right)}^{\alpha }/Q{\left(x\right)}^{\alpha -1}$ for α>1 and use the conventions

(20) $$\begin{array}{c} \frac{0}{0}=0,\;\frac{p}{0}=\infty \;\forall \;p>0. \end{array}$$

For α being zero, one, or infinity, we define by continuous extension of (4)

(21) $$\begin{array}{cc} D_{0}\left(P\|Q\right) & ≜-\log\sum_{x\in \mathrm{supp}(P)}Q\left(x\right), \end{array}$$

(22) $$\begin{array}{cc} D_{1}\left(P\|Q\right) & ≜D(P\|Q), \end{array}$$

(23) $$\begin{array}{cc} D_{\infty }\left(P\|Q\right) & ≜\log\max_{x}\frac{P(x)}{Q(x)}. \end{array}$$

The Rényi divergence for negative α is defined as

(24) $$\begin{array}{c} D_{\alpha }\left(P\|Q\right)≜\frac{1}{\alpha -1}\log\sum_{x}\frac{Q{\left(x\right)}^{1-\alpha }}{P{\left(x\right)}^{-\alpha }}. \end{array}$$

(We use negative α in the proof of Proposition 1 (e) below and in Remark 6. More about negative orders can be found in [8] (Section V). For other applications of negative orders, see [20] (Proof of Theorem 1 and Example 1).)

The Rényi divergence satisfies the following basic properties:

**Proposition** **1.** *Let P and Q be PMFs. Then, the Rényi divergence* $D_{\alpha }\left(P\|Q\right)$ *satisfies the following:*

*(a)*  *For all* α∈[0,∞] *,* $D_{\alpha }\left(P\|Q\right)\geq 0$ *. If* α∈(0,∞] *, then* $D_{\alpha }\left(P\|Q\right)=0$ *if and only if* P=Q *.*
*(b)*  *For all* α∈[0,1) *,* $D_{\alpha }\left(P\|Q\right)$ *is finite if and only if* |supp(P)∩supp(Q)|>0 *. For all* α∈[1,∞] *,* $D_{\alpha }\left(P\|Q\right)$ *is finite if and only if* supp(P)⊆supp(Q) *.*
*(c)*  *The mapping* $\alpha \mapsto D_{\alpha }\left(P\|Q\right)$ *is continuous on* [0,∞] *.*
*(d)*  *The mapping* $\alpha \mapsto D_{\alpha }\left(P\|Q\right)$ *is nondecreasing on* [0,∞] *.*
*(e)*  *The mapping* $\alpha \mapsto \frac{1-\alpha }{\alpha }\;D_{\alpha }\left(P\|Q\right)$ *is nonincreasing on* (0,∞) *.*
*(f)*  *The mapping* $\alpha \mapsto \left(1-\alpha \right)\;D_{\alpha }\left(P\|Q\right)$ *is concave on* [0,∞) *.*
*(g)*  *The mapping* $\alpha \mapsto \left(\alpha -1\right)\;D_{1/\alpha }\left(P\|Q\right)$ *is concave on* (0,∞) *.*
*(h)*  *(Data-processing inequality.) Let* $A_{X^{\prime }|X}$ *be a conditional PMF, and define the PMFs*
  (25) $$\begin{array}{cc} P^{\prime }\left(x^{\prime }\right) & ≜\sum_{x}P\left(x\right)\;A_{X^{\prime }|X}\left(x^{\prime }|x\right), \end{array}$$
  (26) $$\begin{array}{cc} Q^{\prime }\left(x^{\prime }\right) & ≜\sum_{x}Q\left(x\right)\;A_{X^{\prime }|X}\left(x^{\prime }|x\right). \end{array}$$
  *Then, for all* α∈[0,∞] *,*
  (27) $$\begin{array}{c} D_{\alpha }\left(P^{\prime }\|Q^{\prime }\right)\leq D_{\alpha }\left(P\|Q\right). \end{array}$$

**Proof.**  See Appendix A. □

All three conditional Rényi divergences reduce to the unconditional Rényi divergence when both $P_{Y|X}$ and $Q_{Y|X}$ are independent of *X*:

**Remark** **1.** *Let* $P_{Y}$ *,* $Q_{Y}$ *, and* $P_{X}$ *be PMFs. Then, for all* α∈[0,∞] *,*

(28) $$\begin{array}{c} D_{\alpha }^{c}(P_{Y}\|Q_{Y}|P_{X})=D_{\alpha }^{s}(P_{Y}\|Q_{Y}|P_{X})=D_{\alpha }^{l}(P_{Y}\|Q_{Y}\left|P_{X}\right)=D_{\alpha }\left(P_{Y}\|Q_{Y}\right). \end{array}$$

**Proof.**  This follows from the definitions of $D_{\alpha }^{c}\left(\cdot \right)$, $D_{\alpha }^{s}\left(\cdot \right)$, and $D_{\alpha }^{l}\left(\cdot \right)$ in (5)–(7). □

## 3. Csiszár’s Conditional Rényi Divergence

For a PMF $P_{X}$ and conditional PMFs $P_{Y|X}$ and $Q_{Y|X}$, Csiszár’s conditional Rényi divergence $D_{\alpha }^{c}\left(\cdot \right)$ is defined for every α∈[0,∞] as

(29) $$\begin{array}{cc} D_{\alpha }^{c}(P_{Y|X}\|Q_{Y|X}\left|P_{X}\right) & ≜\sum_{x\in \mathrm{supp}(P_{X})}P\left(x\right)\;D_{\alpha }\left(P_{Y|X=x}\|Q_{Y|X=x}\right). \end{array}$$

For α∈(0,1)∪(1,∞),

(30) $$\begin{array}{c} D_{\alpha }^{c}(P_{Y|X}\|Q_{Y|X}\left|P_{X}\right)=\frac{1}{\alpha -1}\sum_{x\in \mathrm{supp}(P_{X})}P\left(x\right)\log\sum_{y}P{\left(y|x\right)}^{\alpha }\;Q{\left(y|x\right)}^{1-\alpha }, \end{array}$$

which follows from the definition of the Rényi divergence in (4). For α being zero, one, or infinity, we obtain from (21)–(23) and (2)

(31) $$\begin{array}{cc} D_{0}^{c}(P_{Y|X}\|Q_{Y|X}\left|P_{X}\right) & =-\sum_{x\in \mathrm{supp}(P_{X})}P\left(x\right)\log\sum_{y\in \mathrm{supp}(P_{Y|X=x})}Q\left(y|x\right), \end{array}$$

(32) $$\begin{array}{cc} D_{1}^{c}(P_{Y|X}\|Q_{Y|X}\left|P_{X}\right) & =D(P_{Y|X}\|Q_{Y|X}|P_{X}), \end{array}$$

(33) $$\begin{array}{cc} D_{\infty }^{c}(P_{Y|X}\|Q_{Y|X}\left|P_{X}\right) & =\sum_{x\in \mathrm{supp}(P_{X})}P\left(x\right)\log\max_{y}\frac{P(y|x)}{Q(y|x)}. \end{array}$$

Augustin [21] and later Csiszár [9] defined the measure of dependence

(34) $$\begin{array}{c} I_{\alpha }^{c}\left(X;Y\right)≜\min_{Q_{Y}}D_{\alpha }^{c}(P_{Y|X}\|Q_{Y}\left|P_{X}\right). \end{array}$$

Augustin used this measure to study the error exponents for channel coding with input constraints, while Csiszár used it to study generalized cutoff rates for channel coding with composition constraints. Nakiboğlu [22] studied more properties of $I_{\alpha }^{c}\left(X;Y\right)$. Inter alia, he analyzed the minimax properties of the Augustin capacity

(35) $$\begin{array}{c} \underset{P_{X}\in A}{\sup}I_{\alpha }^{c}\left(P_{X},P_{Y|X}\right)=\underset{P_{X}\in A}{\sup}\min_{Q_{Y}}D_{\alpha }^{c}(P_{Y|X}\|Q_{Y}\left|P_{X}\right), \end{array}$$

where A⊆P(X) is a constraint set. The Augustin capacity is used in [23] to establish the sphere packing bound for memoryless channels with cost constraints.

The rest of the section presents some properties of $D_{\alpha }^{c}\left(\cdot \right)$. Being an average of Rényi divergences (see (29)), $D_{\alpha }^{c}\left(\cdot \right)$ inherits many properties from the Rényi divergence:

**Proposition** **2.** *Let* $P_{X}$ *be a PMF, and let* $P_{Y|X}$ *and* $Q_{Y|X}$ *be conditional PMFs. Then,*

*(a)*  *For all* α∈[0,∞] *,* $D_{\alpha }^{c}(P_{Y|X}\|Q_{Y|X}\left|P_{X}\right)\geq 0$ *. If* α∈(0,∞] *, then* $D_{\alpha }^{c}(P_{Y|X}\|Q_{Y|X}\left|P_{X}\right)=0$ *if and only if* $(P_{Y|X=x}=Q_{Y|X=x}$ *for all* $x\in \mathrm{supp}(P_{X}))$ *.*
*(b)*  *For all* α∈[0,1) *,* $D_{\alpha }^{c}(P_{Y|X}\|Q_{Y|X}\left|P_{X}\right)$ *is finite if and only if* $(|\mathrm{supp}\left(P_{Y|X=x}\right)\cap \mathrm{supp}\left(Q_{Y|X=x}\right)|>0$ *for all* $x\in \mathrm{supp}(P_{X}))$ *. For all* α∈[1,∞] *,* $D_{\alpha }^{c}(P_{Y|X}\|Q_{Y|X}\left|P_{X}\right)$ *is finite if and only if* $(\mathrm{supp}\left(P_{Y|X=x}\right)\subseteq \mathrm{supp}\left(Q_{Y|X=x}\right)$ *for all* $x\in \mathrm{supp}(P_{X}))$ *.*
*(c)*  *The mapping* $\alpha \mapsto D_{\alpha }^{c}(P_{Y|X}\|Q_{Y|X}\left|P_{X}\right)$ *is continuous on* [0,∞] *.*
*(d)*  *The mapping* $\alpha \mapsto D_{\alpha }^{c}(P_{Y|X}\|Q_{Y|X}\left|P_{X}\right)$ *is nondecreasing on* [0,∞] *.*
*(e)*  *The mapping* $\alpha \mapsto \frac{1-\alpha }{\alpha }\;D_{\alpha }^{c}(P_{Y|X}\|Q_{Y|X}\left|P_{X}\right)$ *is nonincreasing on* (0,∞) *.*
*(f)*  *The mapping* $\alpha \mapsto \left(1-\alpha \right)\;D_{\alpha }^{c}(P_{Y|X}\|Q_{Y|X}\left|P_{X}\right)$ *is concave on* [0,∞) *.*
*(g)*  *The mapping* $\alpha \mapsto \left(\alpha -1\right)\;D_{1/\alpha }^{c}(P_{Y|X}\|Q_{Y|X}\left|P_{X}\right)$ *is concave on* (0,∞) *.*

**Proof.**  These follow from (29) and the properties of the Rényi divergence (Proposition 1). For Parts (f) and (g), recall that a nonnegative weighted sum of concave functions is concave. □

We next consider data-processing inequalities for $D_{\alpha }^{c}\left(\cdot \right)$. We distinguish between processing *Y* and processing *X*. The data-processing inequality for processing *Y* follows from the data-processing inequality for the (unconditional) Rényi divergence:

**Theorem** **1.** *Let* $P_{X}$ *be a PMF, and let* $P_{Y|X}$ *and* $Q_{Y|X}$ *be conditional PMFs. For a conditional PMF* $A_{Y^{\prime }|XY}$ *, define*

(36) $$\begin{array}{cc} P_{Y^{\prime }|X}\left(y^{\prime }|x\right) & ≜\sum_{y}P_{Y|X}\left(y|x\right)\;A_{Y^{\prime }|XY}\left(y^{\prime }|x,y\right), \end{array}$$

(37) $$\begin{array}{cc} Q_{Y^{\prime }|X}\left(y^{\prime }|x\right) & ≜\sum_{y}Q_{Y|X}\left(y|x\right)\;A_{Y^{\prime }|XY}\left(y^{\prime }|x,y\right). \end{array}$$

*Then, for all* α∈[0,∞] *,*

(38) $$\begin{array}{c} D_{\alpha }^{c}(P_{Y^{\prime }|X}\|Q_{Y^{\prime }|X}|P_{X})\leq D_{\alpha }^{c}(P_{Y|X}\|Q_{Y|X}\left|P_{X}\right). \end{array}$$

**Proof.**  See Appendix B. □

The following data-processing inequality for processing *X* holds for α∈[0,1] (as shown in Example 1 below, it does not extend to α∈(1,∞]):

**Theorem** **2.** *Let* $P_{X}$ *be a PMF, and let* $P_{Y|X}$ *and* $Q_{Y|X}$ *be conditional PMFs. For a conditional PMF* $B_{X^{\prime }|X}$ *, define the PMFs*

(39) $$\begin{array}{cc} P_{X^{\prime }}\left(x^{\prime }\right) & ≜\sum_{x}P_{X}\left(x\right)\;B_{X^{\prime }|X}\left(x^{\prime }|x\right), \end{array}$$

(40) $$B_{X|X^{\prime }}\left(x|x^{\prime }\right)≜\left\{\begin{array}{ll} P_{X}\left(x\right)\;B_{X^{\prime }|X}\left(x^{\prime }|x\right)/P_{X^{\prime }}\left(x^{\prime }\right) & if\;P_{X^{\prime }}\left(x^{\prime }\right)>0,\\ 1/|X| & otherwise, \end{array}\right.$$

(41) $$\begin{array}{cc} P_{Y|X^{\prime }}\left(y|x^{\prime }\right) & ≜\sum_{x}B_{X|X^{\prime }}\left(x|x^{\prime }\right)\;P_{Y|X}\left(y|x\right), \end{array}$$

(42) $$\begin{array}{cc} Q_{Y|X^{\prime }}\left(y|x^{\prime }\right) & ≜\sum_{x}B_{X|X^{\prime }}\left(x|x^{\prime }\right)\;Q_{Y|X}\left(y|x\right). \end{array}$$

*Then, for all* α∈[0,1] *,*

(43) $$\begin{array}{c} D_{\alpha }^{c}(P_{Y|X^{\prime }}\|Q_{Y|X^{\prime }}|P_{X^{\prime }})\leq D_{\alpha }^{c}(P_{Y|X}\|Q_{Y|X}\left|P_{X}\right). \end{array}$$

Note that $P_{X^{\prime }}$, $P_{Y|X^{\prime }}$, and $Q_{Y|X^{\prime }}$ in Theorem 2 can be obtained from the following marginalizations:

(44) $$\begin{array}{cc} P_{X^{\prime }}\left(x^{\prime }\right)\;P_{Y|X^{\prime }}\left(y|x^{\prime }\right) & =\sum_{x}P_{X}\left(x\right)\;B_{X^{\prime }|X}\left(x^{\prime }|x\right)\;P_{Y|X}\left(y|x\right), \end{array}$$

(45) $$\begin{array}{cc} P_{X^{\prime }}\left(x^{\prime }\right)\;Q_{Y|X^{\prime }}\left(y|x^{\prime }\right) & =\sum_{x}P_{X}\left(x\right)\;B_{X^{\prime }|X}\left(x^{\prime }|x\right)\;Q_{Y|X}\left(y|x\right). \end{array}$$

**Proof** **of** **Theorem** **2.** See Appendix C. □

As a special case of Theorem 2, we obtain the following relation between the conditional and the unconditional Rényi divergence:

**Corollary** **1.** *For a PMF* $P_{X}$ *and conditional PMFs* $P_{Y|X}$ *and* $Q_{Y|X}$ *, define the marginal PMFs*

(46) $$\begin{array}{cc} P_{Y}\left(y\right) & ≜\sum_{x}P_{X}\left(x\right)\;P_{Y|X}\left(y|x\right), \end{array}$$

(47) $$\begin{array}{cc} Q_{Y}\left(y\right) & ≜\sum_{x}P_{X}\left(x\right)\;Q_{Y|X}\left(y|x\right). \end{array}$$

*Then, for all* α∈[0,1] *,*

(48) $$\begin{array}{c} D_{\alpha }\left(P_{Y}\|Q_{Y}\right)\leq D_{\alpha }^{c}(P_{Y|X}\|Q_{Y|X}\left|P_{X}\right). \end{array}$$

**Proof.**  See Appendix D. □

Consider next α∈(1,∞]. It turns out that Corollary 1, and hence Theorem 2, cannot be extended to these values of α (not even if $Q_{Y|X}$ is restricted to be independent of *X*, i.e., if $Q_{Y|X}=Q_{Y}$):

**Example** **1.** *Let* X=Y={0,1} *. For* ϵ∈(0,1) *, define the PMFs* $P_{X}$ *,* $Q_{Y}^{\left(\epsilon \right)}$ *, and* $P_{Y|X}^{\left(\epsilon \right)}$ *as*

(49) $$\begin{array}{cccc} P_{X}\left(0\right) & =0.5, & \;\;\;P_{X}\left(1\right) & =0.5, \end{array}$$

(50) $$\begin{array}{cccc} Q_{Y}^{\left(\epsilon \right)}\left(0\right) & =1-\epsilon , & \;\;Q_{Y}^{\left(\epsilon \right)}\left(1\right) & =\epsilon , \end{array}$$

(51) $$\begin{array}{cccc} P_{Y|X}^{\left(\epsilon \right)}\left(0|0\right) & =1-\epsilon , & \;P_{Y|X}^{\left(\epsilon \right)}\left(1|0\right) & =\epsilon , \end{array}$$

(52) $$\begin{array}{cccc} P_{Y|X}^{\left(\epsilon \right)}\left(0|1\right) & =\epsilon , & \;\;\;P_{Y|X}^{\left(\epsilon \right)}\left(1|1\right) & =1-\epsilon . \end{array}$$

*Then, for every*α∈(1,∞]*, there exists an*ϵ∈(0,1)*such that*

(53) $$\begin{array}{c} D_{\alpha }\left(P_{Y}\|Q_{Y}^{\left(\epsilon \right)}\right)>D_{\alpha }^{c}\left(P_{Y|X}^{\left(\epsilon \right)}\left\|Q_{Y}^{\left(\epsilon \right)}\right|P_{X}\right), \end{array}$$

*where the PMF*$P_{Y}$*is defined by* (46) *and, irrespective of *ϵ*, satisfies* $P_{Y}\left(0\right)=P_{Y}\left(1\right)=0.5$*.*

**Proof.**  See Appendix E. □

## 4. Sibson’s Conditional Rényi Divergence

For a PMF $P_{X}$ and conditional PMFs $P_{Y|X}$ and $Q_{Y|X}$, Sibson’s conditional Rényi divergence $D_{\alpha }^{s}\left(\cdot \right)$ is defined for every α∈[0,∞] as

(54) $$\begin{array}{cc} D_{\alpha }^{s}(P_{Y|X}\|Q_{Y|X}\left|P_{X}\right) & ≜D_{\alpha }\left(P_{X}P_{Y|X}\|P_{X}Q_{Y|X}\right). \end{array}$$

For α∈(0,1)∪(1,∞),

(55) $$\begin{array}{cc} D_{\alpha }^{s}(P_{Y|X}\|Q_{Y|X}\left|P_{X}\right) & =\frac{1}{\alpha -1}\log\sum_{x\in \mathrm{supp}(P_{X})}P\left(x\right)\sum_{y}P{\left(y|x\right)}^{\alpha }\;Q{\left(y|x\right)}^{1-\alpha } \end{array}$$

(56) $$\begin{array}{cc} \; & =\frac{1}{\alpha -1}\log\sum_{x\in \mathrm{supp}(P_{X})}P\left(x\right)\;2^{\left(\alpha -1\right)D_{\alpha }\left(P_{Y|X=x}\|Q_{Y|X=x}\right)}, \end{array}$$

where (55) and (56) follow from the definition of the Rényi divergence in (4). For α being zero, one, or infinity, we obtain from (21)–(23) and (3)

(57) $$\begin{array}{cc} D_{0}^{s}(P_{Y|X}\|Q_{Y|X}\left|P_{X}\right) & =-\log\sum_{x\in \mathrm{supp}(P_{X})}P\left(x\right)\sum_{y\in \mathrm{supp}(P_{Y|X=x})}Q\left(y|x\right), \end{array}$$

(58) $$\begin{array}{cc} D_{1}^{s}(P_{Y|X}\|Q_{Y|X}\left|P_{X}\right) & =D(P_{Y|X}\|Q_{Y|X}|P_{X}), \end{array}$$

(59) D∞s(PY|X‖QY|X|PX)=logmaxx∈supp(PX)maxy∈supp(PX)P(y|x)Q(y|x).

Sibson [10] defined the measure of dependence

(60) $$\begin{array}{c} I_{\alpha }^{s}\left(X;Y\right)≜\min_{Q_{Y}}D_{\alpha }^{s}(P_{Y|X}\|Q_{Y}\left|P_{X}\right). \end{array}$$

This minimum can be computed explicitly [10] (Corollary 2.3): For α∈(0,1)∪(1,∞),

(61) $$\begin{array}{c} I_{\alpha }^{s}\left(X;Y\right)=\frac{\alpha }{\alpha -1}\log\sum_{y}{\left[\sum_{x}P\left(x\right)\;P{\left(y|x\right)}^{\alpha }\right]}^{\frac{1}{\alpha }}, \end{array}$$

and for α being one or infinity,

(62) $$\begin{array}{cc} I_{1}^{s}\left(X;Y\right) & =I(X;Y), \end{array}$$

(63) $$\begin{array}{cc} I_{\infty }^{s}\left(X;Y\right) & =\log\sum_{y}\max_{x}P\left(y|x\right), \end{array}$$

where I(X;Y) denotes Shannon’s mutual information.

The concavity and convexity properties of $D_{\alpha }^{s}\left(\cdot \right)$ and $I_{\alpha }^{s}\left(X;Y\right)$ were studied by Ho–Verdú [24]. More properties of $I_{\alpha }^{s}\left(X;Y\right)$ were collected by Verdú [25]. The maximization of $I_{\alpha }^{s}\left(X;Y\right)$ with respect to $P_{X}$ and the minimax properties of $D_{\alpha }^{s}\left(\cdot \right)$ were studied by Nakiboğlu [26] and Cai–Verdú [27].

The conditional Rényi divergence $D_{\alpha }^{s}\left(\cdot \right)$ was used by Fong and Tan [28] to establish strong converse theorems for multicast networks. Yu and Tan [29] analyzed channel resolvability, among other measures, in terms of $D_{\alpha }^{s}\left(\cdot \right)$.

From (61) we see that Gallager’s $E_{0}$ function [11], which is defined as

(64) $$\begin{array}{c} E_{0}\left(\rho ,P_{X},P_{Y|X}\right)≜-\log\sum_{y}{\left[\sum_{x}P\left(x\right)\;P{\left(y|x\right)}^{\frac{1}{1+\rho }}\right]}^{1+\rho }, \end{array}$$

is in one-to-one correspondence to Sibson’s measure of dependence:

(65) $$\begin{array}{c} I_{\alpha }^{s}\left(X;Y\right)=\frac{\alpha }{1-\alpha }\;E_{0}\left(\frac{1-\alpha }{\alpha },P_{X},P_{Y|X}\right). \end{array}$$

Gallager’s $E_{0}$ function is important in channel coding: it appears in the random coding exponent [30] and in the sphere packing exponent [31,32] (see also Gallager [11]). The exponential strong converse theorem proved by Arimoto [33] also uses the $E_{0}$ function. Polyanskiy and Verdú [34] extended the exponential strong converse theorem to channels with feedback. Augustin [21] and Nakiboğlu [35,36] extended the sphere packing bound to channels with feedback.

The rest of the section presents some properties of $D_{\alpha }^{s}\left(\cdot \right)$. Because $D_{\alpha }^{s}\left(\cdot \right)$ can be written as an (unconditional) Rényi divergence (see (54)), it inherits many properties from the Rényi divergence:

**Proposition** **3.** *Let* $P_{X}$ *be a PMF, and let* $P_{Y|X}$ *and* $Q_{Y|X}$ *be conditional PMFs. Then,*

*(a)*  *For all* α∈[0,∞] *,* $D_{\alpha }^{s}(P_{Y|X}\|Q_{Y|X}\left|P_{X}\right)\geq 0$ *. If* α∈(0,∞] *, then* $D_{\alpha }^{s}(P_{Y|X}\|Q_{Y|X}\left|P_{X}\right)=0$ *if and only if* $(P_{Y|X=x}=Q_{Y|X=x}$ *for all* $x\in \mathrm{supp}(P_{X}))$ *.*
*(b)*  *For all* α∈[0,1) *,* $D_{\alpha }^{s}(P_{Y|X}\|Q_{Y|X}\left|P_{X}\right)$ *is finite if and only if (there exists an* $x\in \mathrm{supp}(P_{X})$ *such that* $\left|\mathrm{supp}\left(P_{Y|X=x}\right)\cap \mathrm{supp}\left(Q_{Y|X=x}\right)|>0\right)$ *. For all* α∈[1,∞] *,* $D_{\alpha }^{s}(P_{Y|X}\|Q_{Y|X}\left|P_{X}\right)$ *is finite if and only if* $(\mathrm{supp}\left(P_{Y|X=x}\right)\subseteq \mathrm{supp}\left(Q_{Y|X=x}\right)$ *for all* $x\in \mathrm{supp}(P_{X}))$ *.*
*(c)*  *The mapping* $\alpha \mapsto D_{\alpha }^{s}(P_{Y|X}\|Q_{Y|X}\left|P_{X}\right)$ *is continuous on* [0,∞] *.*
*(d)*  *The mapping* $\alpha \mapsto D_{\alpha }^{s}(P_{Y|X}\|Q_{Y|X}\left|P_{X}\right)$ *is nondecreasing on* [0,∞] *.*
*(e)*  *The mapping* $\alpha \mapsto \frac{1-\alpha }{\alpha }\;D_{\alpha }^{s}(P_{Y|X}\|Q_{Y|X}\left|P_{X}\right)$ *is nonincreasing on* (0,∞) *.*
*(f)*  *The mapping* $\alpha \mapsto \left(1-\alpha \right)\;D_{\alpha }^{s}(P_{Y|X}\|Q_{Y|X}\left|P_{X}\right)$ *is concave on* [0,∞) *.*
*(g)*  *The mapping* $\alpha \mapsto \left(\alpha -1\right)\;D_{1/\alpha }^{s}(P_{Y|X}\|Q_{Y|X}\left|P_{X}\right)$ *is concave on* (0,∞) *.*

**Proof.**  These follow from (54) and the properties of the Rényi divergence (Proposition 1). □

We next consider data-processing inequalities for $D_{\alpha }^{s}\left(\cdot \right)$. We distinguish between processing *Y* and processing *X*. The data-processing inequality for processing *Y* follows from the data-processing inequality for the (unconditional) Rényi divergence:

**Theorem** **3.** *Let* $P_{X}$ *be a PMF, and let* $P_{Y|X}$ *and* $Q_{Y|X}$ *be conditional PMFs. For a conditional PMF* $A_{Y^{\prime }|XY}$ *, define*

(66) $$\begin{array}{cc} P_{Y^{\prime }|X}\left(y^{\prime }|x\right) & ≜\sum_{y}P_{Y|X}\left(y|x\right)\;A_{Y^{\prime }|XY}\left(y^{\prime }|x,y\right), \end{array}$$

(67) $$\begin{array}{cc} Q_{Y^{\prime }|X}\left(y^{\prime }|x\right) & ≜\sum_{y}Q_{Y|X}\left(y|x\right)\;A_{Y^{\prime }|XY}\left(y^{\prime }|x,y\right). \end{array}$$

*Then, for all* α∈[0,∞] *,*

(68) $$\begin{array}{c} D_{\alpha }^{s}(P_{Y^{\prime }|X}\|Q_{Y^{\prime }|X}|P_{X})\leq D_{\alpha }^{s}(P_{Y|X}\|Q_{Y|X}\left|P_{X}\right). \end{array}$$

**Proof.**  See Appendix F. □

The data-processing inequality for processing *X* similarly follows from the data-processing inequality for the (unconditional) Rényi divergence:

**Theorem** **4.** *Let* $P_{X}$ *be a PMF, and let* $P_{Y|X}$ *and* $Q_{Y|X}$ *be conditional PMFs. For a conditional PMF* $B_{X^{\prime }|X}$ *, define the PMFs*

(69) $$\begin{array}{cc} P_{X^{\prime }}\left(x^{\prime }\right) & ≜\sum_{x}P_{X}\left(x\right)\;B_{X^{\prime }|X}\left(x^{\prime }|x\right), \end{array}$$

(70) $$\begin{array}{cc} B_{X|X^{\prime }}\left(x|x^{\prime }\right) & ≜\left\{\begin{array}{cc} P_{X}\left(x\right)\;B_{X^{\prime }|X}\left(x^{\prime }|x\right)/P_{X^{\prime }}\left(x^{\prime }\right) & if\;P_{X^{\prime }}\left(x^{\prime }\right)>0,\\ 1/|X| & otherwise, \end{array}\right. \end{array}$$

(71) $$\begin{array}{cc} P_{Y|X^{\prime }}\left(y|x^{\prime }\right) & ≜\sum_{x}B_{X|X^{\prime }}\left(x|x^{\prime }\right)\;P_{Y|X}\left(y|x\right), \end{array}$$

(72) $$\begin{array}{cc} Q_{Y|X^{\prime }}\left(y|x^{\prime }\right) & ≜\sum_{x}B_{X|X^{\prime }}\left(x|x^{\prime }\right)\;Q_{Y|X}\left(y|x\right). \end{array}$$

*Then, for all* α∈[0,∞] *,*

(73) $$\begin{array}{c} D_{\alpha }^{s}(P_{Y|X^{\prime }}\|Q_{Y|X^{\prime }}|P_{X^{\prime }})\leq D_{\alpha }^{s}(P_{Y|X}\|Q_{Y|X}\left|P_{X}\right). \end{array}$$

**Proof.**  See Appendix G. □

As a special case of Theorem 4, we obtain the following relation between the conditional and the unconditional Rényi divergence:

**Corollary** **2.** *Let* $P_{X}$ *be a PMF, and let* $P_{Y|X}$ *and* $Q_{Y|X}$ *be conditional PMFs. Define the marginal PMFs*

(74) $$\begin{array}{cc} P_{Y}\left(y\right) & ≜\sum_{x}P_{X}\left(x\right)\;P_{Y|X}\left(y|x\right), \end{array}$$

(75) $$\begin{array}{cc} Q_{Y}\left(y\right) & ≜\sum_{x}P_{X}\left(x\right)\;Q_{Y|X}\left(y|x\right). \end{array}$$

*Then, for all* α∈[0,∞] *,*

(76) $$\begin{array}{c} D_{\alpha }\left(P_{Y}\|Q_{Y}\right)\leq D_{\alpha }^{s}(P_{Y|X}\|Q_{Y|X}\left|P_{X}\right). \end{array}$$

**Proof.**  This follows from Theorem 4 in the same way that Corollary 1 followed from Theorem 2. □

## 5. New Conditional Rényi Divergence

Let $P_{X}$ be a PMF, and let $P_{Y|X}$ and $Q_{Y|X}$ be conditional PMFs. For α∈(0,1)∪(1,∞), define

(77) $$\begin{array}{cc} D_{\alpha }^{l}(P_{Y|X}\|Q_{Y|X}\left|P_{X}\right) & ≜\frac{\alpha }{\alpha -1}\log\sum_{x\in \mathrm{supp}(P_{X})}P\left(x\right)\;2^{\frac{\alpha -1}{\alpha }D_{\alpha }\left(P_{Y|X=x}\|Q_{Y|X=x}\right)} \end{array}$$

(78) $$\begin{array}{cc} \; & =\frac{\alpha }{\alpha -1}\log\sum_{x\in \mathrm{supp}(P_{X})}P\left(x\right)\;{\left[\sum_{y}P{\left(y|x\right)}^{\alpha }\;Q{\left(y|x\right)}^{1-\alpha }\right]}^{\frac{1}{\alpha }}, \end{array}$$

where (78) follows from the definition of the Rényi divergence in (4). (Except for the sign, the exponential averaging in (77) is very similar to the one of the Arimoto–Rényi conditional entropy; compare with (147) below.) For α being zero, one, or infinity, we define by continuous extension of (77)

(79) $$\begin{array}{cc} D_{0}^{l}(P_{Y|X}\|Q_{Y|X}\left|P_{X}\right) & ≜-\log\max_{x\in \mathrm{supp}(P_{X})}\sum_{y\in \mathrm{supp}(P_{Y|X=x})}Q\left(y|x\right), \end{array}$$

(80) $$\begin{array}{cc} D_{1}^{l}(P_{Y|X}\|Q_{Y|X}\left|P_{X}\right) & ≜D(P_{Y|X}\|Q_{Y|X}|P_{X}), \end{array}$$

(81) $$\begin{array}{cc} D_{\infty }^{l}(P_{Y|X}\|Q_{Y|X}\left|P_{X}\right) & ≜\log\sum_{x\in \mathrm{supp}(P_{X})}P\left(x\right)\max_{y}\frac{P(y|x)}{Q(y|x)}. \end{array}$$

This conditional Rényi divergence has an operational meaning in horse betting with side information (see Theorem 10 below). Before discussing the measure of dependence associated with $D_{\alpha }^{l}\left(\cdot \right)$, we establish the following alternative characterization of $D_{\alpha }^{l}\left(\cdot \right)$:

**Proposition** **4.** *Let* $P_{X}$ *be a PMF, and let* $P_{Y|X}$ *and* $Q_{Y|X}$ *be conditional PMFs. Then, for all* α∈[0,∞] *,*

(82) $$\begin{array}{c} D_{\alpha }^{l}(P_{Y|X}\|Q_{Y|X}\left|P_{X}\right)=\min_{Q_{X}}D_{\alpha }\left(P_{X}P_{Y|X}\|Q_{X}Q_{Y|X}\right). \end{array}$$

**Proof.**  We first treat the case α∈(0,1)∪(1,∞). Some algebra reveals that, for every PMF $Q_{X}$,

(83) $$\begin{array}{c} D_{\alpha }\left(P_{X}P_{Y|X}\|Q_{X}Q_{Y|X}\right)=D_{\alpha }\left(Q_{X}^{*(\alpha )}\|Q_{X}\right)+\frac{\alpha }{\alpha -1}\log\sum_{x\in \mathrm{supp}(P_{X})}P\left(x\right)\;{\left[\sum_{y}P{\left(y|x\right)}^{\alpha }\;Q{\left(y|x\right)}^{1-\alpha }\right]}^{\frac{1}{\alpha }}, \end{array}$$

where the PMF $Q_{X}^{*(\alpha )}$ is defined as

(84) $$\begin{array}{c} Q_{X}^{*(\alpha )}\left(x\right)≜\frac{P\left(x\right)\;{\left[\sum_{y}P{\left(y|x\right)}^{\alpha }\;Q{\left(y|x\right)}^{1-\alpha }\right]}^{1/\alpha }}{\sum_{x^{\prime }\in \mathrm{supp}\left(P_{X}\right)}P\left(x^{\prime }\right)\;{\left[\sum_{y}P{\left(y|x^{\prime }\right)}^{\alpha }\;Q{\left(y|x^{\prime }\right)}^{1-\alpha }\right]}^{1/\alpha }}. \end{array}$$

The right-hand side (RHS) of (82) is thus equal to the minimum over $Q_{X}$ of the RHS of (83). Since $D_{\alpha }\left(Q_{X}^{*(\alpha )}\|Q_{X}\right)\geq 0$ with equality if $Q_{X}=Q_{X}^{*(\alpha )}$ (Proposition 1 (a)), this minimum is equal to the second term on the RHS of (83), which, by (78), equals $D_{\alpha }^{l}(P_{Y|X}\|Q_{Y|X}\left|P_{X}\right)$.

For α=1 and α=∞, (82) follows from the same argument using that, for every PMF $Q_{X}$,

(85) $$\begin{array}{cc} D_{1}\left(P_{X}P_{Y|X}\|Q_{X}Q_{Y|X}\right) & =D\left(P_{X}\|Q_{X}\right)+D(P_{Y|X}\|Q_{Y|X}\left|P_{X}\right), \end{array}$$

(86) $$\begin{array}{cc} D_{\infty }\left(P_{X}P_{Y|X}\|Q_{X}Q_{Y|X}\right) & =D_{\infty }\left(Q_{X}^{*(\infty )}\|Q_{X}\right)+\log\sum_{x\in \mathrm{supp}(P_{X})}P\left(x\right)\max_{y}\frac{P(y|x)}{Q(y|x)}, \end{array}$$

where the PMF $Q_{X}^{*(\infty )}$ is defined as

(87) $$\begin{array}{c} Q_{X}^{*(\infty )}\left(x\right)≜\frac{P\left(x\right)\max_{y}\left[P(y|x)/Q(y|x)\right]}{\sum_{x^{\prime }\in \mathrm{supp}\left(P_{X}\right)}P\left(x^{\prime }\right)\max_{y}\left[P\left(y|x^{\prime }\right)/Q\left(y|x^{\prime }\right)\right]}. \end{array}$$

For α=0, (82) holds because

(88) $$\begin{array}{cc} \min_{Q_{X}}D_{0}\left(P_{X}P_{Y|X}\|Q_{X}Q_{Y|X}\right) & =\min_{Q_{X}}-\log\sum_{x\in \mathrm{supp}(P_{X})}Q\left(x\right)\sum_{y\in \mathrm{supp}(P_{Y|X=x})}Q\left(y|x\right) \end{array}$$

(89) $$\begin{array}{cc} \; & =-\log\max_{Q_{X}}\sum_{x\in \mathrm{supp}(P_{X})}Q\left(x\right)\sum_{y\in \mathrm{supp}(P_{Y|X=x})}Q\left(y|x\right) \end{array}$$

(90) $$\begin{array}{cc} \; & =-\log\max_{x\in \mathrm{supp}(P_{X})}\sum_{y\in \mathrm{supp}(P_{Y|X=x})}Q\left(y|x\right) \end{array}$$

(91) $$\begin{array}{cc} \; & =D_{0}^{l}(P_{Y|X}\|Q_{Y|X}\left|P_{X}\right), \end{array}$$

where (88) follows from the definition of $D_{0}\left(P\|Q\right)$ in (21), and (91) follows from (79). □

Tomamichel and Hayashi [37] and Lapidoth and Pfister [3] independently introduced and studied the dependence measure

(92) $$\begin{array}{cc} J_{\alpha }\left(X;Y\right) & ≜\min_{Q_{X},\;Q_{Y}}D_{\alpha }\left(P_{XY}\|Q_{X}Q_{Y}\right). \end{array}$$

(For some measure-theoretic properties of $J_{\alpha }\left(X;Y\right)$, see Aishwarya–Madiman [38].) The measure $J_{\alpha }\left(X;Y\right)$ can be related to the error exponents in a hypothesis testing problem where the samples are either from a known joint distribution or an unknown product distribution (see [37] (Equation (57)) and [39]). It also appears in horse betting with side information (see Theorem 11 below).

Similar to $I_{\alpha }^{c}\left(X;Y\right)$ in (34) and $I_{\alpha }^{s}\left(X;Y\right)$ in (60), the measure $J_{\alpha }\left(X;Y\right)$ can be expressed as a minimization involving the new conditional Rényi divergence:

**Proposition** **5.** *Let* $P_{XY}$ *be a joint PMF. Denote its marginal PMFs by* $P_{X}$ *and* $P_{Y}$ *and its conditional PMFs by* $P_{Y|X}$ *and* $P_{X|Y}$ *, so* $P_{XY}=P_{X}P_{Y|X}=P_{Y}P_{X|Y}$ *. Then, for all* α∈[0,∞] *,*

(93) $$\begin{array}{cc} J_{\alpha }\left(X;Y\right) & =\min_{Q_{Y}}D_{\alpha }^{l}(P_{Y|X}\|Q_{Y}\left|P_{X}\right) \end{array}$$

(94) $$\begin{array}{cc} \; & =\min_{Q_{X}}D_{\alpha }^{l}(P_{X|Y}\|Q_{X}\left|P_{Y}\right). \end{array}$$

**Proof.**  Equation (93) holds because

(95) $$\begin{array}{cc} \min_{Q_{Y}}D_{\alpha }^{l}(P_{Y|X}\|Q_{Y}\left|P_{X}\right) & =\min_{Q_{Y}}\min_{Q_{X}}D_{\alpha }\left(P_{X}P_{Y|X}\|Q_{X}Q_{Y}\right) \end{array}$$

(96) $$\begin{array}{cc} \; & =J_{\alpha }\left(X;Y\right), \end{array}$$

where (95) follows from Proposition 4, and (96) follows from (92). Swapping the roles of *X* and *Y* establishes (94):

(97) $$\begin{array}{cc} \min_{Q_{X}}D_{\alpha }^{l}(P_{X|Y}\|Q_{X}\left|P_{Y}\right) & =\min_{Q_{X}}\min_{Q_{Y}}D_{\alpha }\left(P_{Y}P_{X|Y}\|Q_{Y}Q_{X}\right) \end{array}$$

(98) $$\begin{array}{cc} \; & =J_{\alpha }\left(X;Y\right), \end{array}$$

where (97) follows from Proposition 4, and (98) follows from (92). □

The rest of the section presents some properties of $D_{\alpha }^{l}\left(\cdot \right)$.

**Proposition** **6.** *Let* $P_{X}$ *be a PMF, and let* $P_{Y|X}$ *and* $Q_{Y|X}$ *be conditional PMFs. Then,*

*(a)*  *For all* α∈[0,∞] *,* $D_{\alpha }^{l}(P_{Y|X}\|Q_{Y|X}\left|P_{X}\right)\geq 0$ *. If* α∈(0,∞] *, then* $D_{\alpha }^{l}(P_{Y|X}\|Q_{Y|X}\left|P_{X}\right)=0$ *if and only if* $(P_{Y|X=x}=Q_{Y|X=x}$ *for all* $x\in \mathrm{supp}(P_{X}))$ *.*
*(b)*  *For all* α∈[0,1) *,* $D_{\alpha }^{l}(P_{Y|X}\|Q_{Y|X}\left|P_{X}\right)$ *is finite if and only if (there exists an* $x\in \mathrm{supp}(P_{X})$ *such that* $\left|\mathrm{supp}\left(P_{Y|X=x}\right)\cap \mathrm{supp}\left(Q_{Y|X=x}\right)|>0\right)$ *. For all* α∈[1,∞] *,* $D_{\alpha }^{l}(P_{Y|X}\|Q_{Y|X}\left|P_{X}\right)$ *is finite if and only if* $(\mathrm{supp}\left(P_{Y|X=x}\right)\subseteq \mathrm{supp}\left(Q_{Y|X=x}\right)$ *for all* $x\in \mathrm{supp}(P_{X}))$ *.*
*(c)*  *The mapping* $\alpha \mapsto D_{\alpha }^{l}(P_{Y|X}\|Q_{Y|X}\left|P_{X}\right)$ *is continuous on* [0,∞] *.*
*(d)*  *The mapping* $\alpha \mapsto D_{\alpha }^{l}(P_{Y|X}\|Q_{Y|X}\left|P_{X}\right)$ *is nondecreasing on* [0,∞] *.*
*(e)*  *The mapping* $\alpha \mapsto \frac{1-\alpha }{\alpha }\;D_{\alpha }^{l}(P_{Y|X}\|Q_{Y|X}\left|P_{X}\right)$ *is nonincreasing on* (0,∞) *.*
*(f)*  *The mapping $\alpha \mapsto \left(1-\alpha \right)\;D_{\alpha }^{l}(P_{Y|X}\|Q_{Y|X}\left|P_{X}\right)$ is concave on [0,1].*
*(g)*  *The mapping* $\alpha \mapsto \left(\alpha -1\right)\;D_{1/\alpha }^{l}(P_{Y|X}\|Q_{Y|X}\left|P_{X}\right)$ *is concave on* [1,∞) *.*

**Proof.**  We prove these properties as follows:

(a) For all α∈[0,∞], Proposition 4 implies
  (99) $$\begin{array}{c} D_{\alpha }^{l}(P_{Y|X}\|Q_{Y|X}\left|P_{X}\right)=\min_{Q_{X}}D_{\alpha }\left(P_{X}P_{Y|X}\|Q_{X}Q_{Y|X}\right). \end{array}$$
  The nonnegativity of $D_{\alpha }^{l}\left(\cdot \right)$ now follows from the nonnegativity of the Rényi divergence (Proposition 1 (a)). If $(P_{Y|X=x}=Q_{Y|X=x}$ for all $x\in \mathrm{supp}(P_{X}))$, then $P_{X}P_{Y|X}=P_{X}Q_{Y|X}$. Hence, using $Q_{X}=P_{X}$ on the RHS of (99), $D_{\alpha }^{l}(P_{Y|X}\|Q_{Y|X}\left|P_{X}\right)$ equals zero. Conversely, if α∈(0,∞] and $D_{\alpha }^{l}\left(\cdot \right)=0$, then $P_{X}P_{Y|X}=Q_{X}Q_{Y|X}$ for some $Q_{X}$ by Proposition 1 (a), which implies $(P_{Y|X=x}=Q_{Y|X=x}$ for all $x\in \mathrm{supp}(P_{X}))$.
(b) This follows from the definitions in (77) and (79)–(81) and the conventions in (20).
(c) For α∈(0,1)∪(1,∞), $D_{\alpha }^{l}\left(\cdot \right)$ is continuous because it is, by its definition in (77), a composition of continuous functions. The continuity at α=1 follows from a careful application of L’Hôpital’s rule.
  We next consider the continuity at α=0. Define $\tau ≜\min_{x\in \mathrm{supp}(P_{X})}P\left(x\right)$. Then, for all α∈(0,1),
  (100) $$\begin{array}{cc} \left(\alpha -1\right)\;D_{\alpha }^{l}(P_{Y|X}\|Q_{Y|X}\left|P_{X}\right) & =\alpha \log\sum_{x\in \mathrm{supp}(P_{X})}P\left(x\right)\;2^{\frac{\alpha -1}{\alpha }D_{\alpha }\left(P_{Y|X=x}\|Q_{Y|X=x}\right)} \end{array}$$
  (101) $$\begin{array}{cc} \; & \geq \alpha \log\sum_{x\in \mathrm{supp}(P_{X})}\tau \;2^{\frac{\alpha -1}{\alpha }D_{\alpha }\left(P_{Y|X=x}\|Q_{Y|X=x}\right)} \end{array}$$
  (102) $$\begin{array}{cc} \; & \geq \alpha \log\max_{x\in \mathrm{supp}(P_{X})}\tau \;2^{\frac{\alpha -1}{\alpha }D_{\alpha }\left(P_{Y|X=x}\|Q_{Y|X=x}\right)} \end{array}$$
  (103) $$\begin{array}{cc} \; & =\alpha \log\tau +\max_{x\in \mathrm{supp}(P_{X})}\left(\alpha -1\right)\;D_{\alpha }\left(P_{Y|X=x}\|Q_{Y|X=x}\right), \end{array}$$
  where (100) follows from the definition in (77). On the other hand, for all α∈(0,1),
  (104) $$\begin{array}{cc} \left(\alpha -1\right)\;D_{\alpha }^{l}(P_{Y|X}\|Q_{Y|X}\left|P_{X}\right) & =\alpha \log\sum_{x\in \mathrm{supp}(P_{X})}P\left(x\right)\;2^{\frac{\alpha -1}{\alpha }D_{\alpha }\left(P_{Y|X=x}\|Q_{Y|X=x}\right)} \end{array}$$
  (105) $$\begin{array}{cc} \; & \leq \alpha \log\max_{x\in \mathrm{supp}(P_{X})}2^{\frac{\alpha -1}{\alpha }D_{\alpha }\left(P_{Y|X=x}\|Q_{Y|X=x}\right)} \end{array}$$
  (106) $$\begin{array}{cc} \; & =\max_{x\in \mathrm{supp}(P_{X})}\left(\alpha -1\right)\;D_{\alpha }\left(P_{Y|X=x}\|Q_{Y|X=x}\right). \end{array}$$
  Because $\lim_{\alpha \to 0}\alpha \log\tau =0$, it follows from (103) and (106) and the sandwich theorem that
  (107) $$\begin{array}{cc} \lim_{\alpha ↓0}D_{\alpha }^{l}(P_{Y|X}\|Q_{Y|X}\left|P_{X}\right) & =\lim_{\alpha ↓0}\frac{1}{\alpha -1}\max_{x\in \mathrm{supp}(P_{X})}\left(\alpha -1\right)\;D_{\alpha }\left(P_{Y|X=x}\|Q_{Y|X=x}\right) \end{array}$$
  (108) $$\begin{array}{cc} \; & =-\log\max_{x\in \mathrm{supp}(P_{X})}\sum_{y\in \mathrm{supp}(P_{Y|X=x})}Q\left(y|x\right), \end{array}$$
  where (108) follows from the continuity of the Rényi divergence (Proposition 1 (c)) and the definition of $D_{0}\left(P\|Q\right)$ in (21).
  We conclude with the continuity at α=∞. Observe that
  (109) $$\begin{array}{cc} \lim_{\alpha \to \infty }D_{\alpha }^{l}(P_{Y|X}\|Q_{Y|X}\left|P_{X}\right) & =\lim_{\alpha \to \infty }\frac{\alpha }{\alpha -1}\log\sum_{x\in \mathrm{supp}(P_{X})}P\left(x\right)\;2^{\frac{\alpha -1}{\alpha }D_{\alpha }\left(P_{Y|X=x}\|Q_{Y|X=x}\right)} \end{array}$$
  (110) $$\begin{array}{cc} \; & =\log\sum_{x\in \mathrm{supp}(P_{X})}P\left(x\right)\;2^{\lim_{\alpha \to \infty }D_{\alpha }\left(P_{Y|X=x}\|Q_{Y|X=x}\right)} \end{array}$$
  (111) $$\begin{array}{cc} \; & =\log\sum_{x\in \mathrm{supp}(P_{X})}P\left(x\right)\max_{y}\frac{P(y|x)}{Q(y|x)}, \end{array}$$
  where (109) follows from the definition in (77), and (111) follows from the continuity of the Rényi divergence (Proposition 1 (c)) and the definition of $D_{\infty }\left(P\|Q\right)$ in (23).
(d) For all α∈[0,∞], Proposition 4 implies
  (112) $$\begin{array}{c} D_{\alpha }^{l}(P_{Y|X}\|Q_{Y|X}\left|P_{X}\right)=\min_{Q_{X}}D_{\alpha }\left(P_{X}P_{Y|X}\|Q_{X}Q_{Y|X}\right). \end{array}$$
  Because $\alpha \mapsto D_{\alpha }\left(P\|Q\right)$ is nonincreasing on [0,∞] (Proposition 1 (d)) and because the pointwise minimum preserves the monotonicity, the mapping $\alpha \mapsto D_{\alpha }^{l}\left(\cdot \right)$ is nonincreasing on [0,∞].
(e) By Proposition 4,
  (113) 1−ααDαl(PY|X‖QY|X|PX)=minQXXYZ1−ααDα(PXXYZPY|XXYZ‖QXXYZQY|XXYZ)ifα∈(0,1],maxQXXYZ1−ααDα(PXXYZPY|XXYZ‖QXXYZQY|XXYZ)ifα∈(1,∞).
  By the nonnegativity of the Rényi divergence (Proposition 1 (a)), the RHS of (113) is nonnegative for α∈(0,1] and nonpositive for α∈(1,∞). Hence, it suffices to show separately that the mapping $\alpha \mapsto \frac{1-\alpha }{\alpha }\;D_{\alpha }^{l}(P_{Y|X}\|Q_{Y|X}\left|P_{X}\right)$ is nonincreasing on (0,1] and on (1,∞). This is indeed the case: the mapping $\alpha \mapsto \frac{1-\alpha }{\alpha }\;D_{\alpha }\left(P_{X}P_{Y|X}\|Q_{X}Q_{Y|X}\right)$ on the RHS of (113) is nonincreasing on (0,∞) (Proposition 1 (e)), and the monotonicity is preserved by the pointwise minimum and maximum, respectively.
(f) For α∈[0,1], Proposition 4 implies that
  (114) $$\begin{array}{cc} \left(1-\alpha \right)\;D_{\alpha }^{l}(P_{Y|X}\|Q_{Y|X}\left|P_{X}\right) & =\min_{Q_{X}}\left[\left(1-\alpha \right)\;D_{\alpha }\left(P_{X}P_{Y|X}\|Q_{X}Q_{Y|X}\right)\right]. \end{array}$$
  Because $\alpha \mapsto \left(1-\alpha \right)\;D_{\alpha }\left(P_{X}P_{Y|X}\|Q_{X}Q_{Y|X}\right)$ is concave on [0,1] (Proposition 1 (f)) and because the pointwise minimum preserves the concavity, the mapping $\alpha \mapsto \left(1-\alpha \right)\;D_{\alpha }^{l}(P_{Y|X}\|Q_{Y|X}\left|P_{X}\right)$ is concave on [0,1].
(g) This follows from Proposition 1 (g) in the same way that Part (f) followed from Proposition 1 (f). □

We next consider data-processing inequalities for $D_{\alpha }^{l}\left(\cdot \right)$. We distinguish between processing *Y* and processing *X*. The data-processing inequality for processing *Y* follows from the data-processing inequality for the (unconditional) Rényi divergence:

**Theorem** **5.** *Let $P_{X}$ be a PMF, and let $P_{Y|X}$ and $Q_{Y|X}$ be conditional PMFs. For a conditional PMF $A_{Y^{\prime }|XY}$, define*

(115) $$\begin{array}{cc} P_{Y^{\prime }|X}\left(y^{\prime }|x\right) & ≜\sum_{y}P_{Y|X}\left(y|x\right)\;A_{Y^{\prime }|XY}\left(y^{\prime }|x,y\right), \end{array}$$

(116) $$\begin{array}{cc} Q_{Y^{\prime }|X}\left(y^{\prime }|x\right) & ≜\sum_{y}Q_{Y|X}\left(y|x\right)\;A_{Y^{\prime }|XY}\left(y^{\prime }|x,y\right). \end{array}$$

*Then, for all* α∈[0,∞] *,*

(117) $$\begin{array}{c} D_{\alpha }^{l}(P_{Y^{\prime }|X}\|Q_{Y^{\prime }|X}|P_{X})\leq D_{\alpha }^{l}(P_{Y|X}\|Q_{Y|X}\left|P_{X}\right). \end{array}$$

**Proof.**  We prove (117) for α∈(0,1)∪(1,∞); the claim will then extend to α∈[0,∞] by the continuity of $D_{\alpha }^{l}\left(\cdot \right)$ in α (Proposition 6 (c)). For every $x\in \mathrm{supp}(P_{X})$, we can apply Proposition 1 (h) with the substitution of $A_{Y^{\prime }|Y,X=x}$ for $A_{Y^{\prime }|Y}$ to obtain

(118) $$\begin{array}{cc} D_{\alpha }\left(P_{Y^{\prime }|X=x}\|Q_{Y^{\prime }|X=x}\right) & \leq D_{\alpha }\left(P_{Y|X=x}\|Q_{Y|X=x}\right). \end{array}$$

For α∈(0,1)∪(1,∞), (117) now follows from (77) and (118). □

Processing *X* is different. Consider first $Q_{Y|X}$ that does not depend on *X*. Then, writing $Q_{Y|X}=Q_{Y}$, we have the following result (which, as shown in Example 2 below, does not extend to general $Q_{Y|X}$):

**Theorem** **6.** *Let* $P_{X}$ *and* $Q_{Y}$ *be PMFs, and let* $P_{Y|X}$ *be a conditional PMF. For a conditional PMF* $B_{X^{\prime }|X}$ *, define the PMFs*

(119) $$\begin{array}{cc} P_{X^{\prime }}\left(x^{\prime }\right) & ≜\sum_{x}P_{X}\left(x\right)\;B_{X^{\prime }|X}\left(x^{\prime }|x\right), \end{array}$$

(120) $$\begin{array}{cc} B_{X|X^{\prime }}\left(x|x^{\prime }\right) & ≜\left\{\begin{array}{cc} P_{X}\left(x\right)\;B_{X^{\prime }|X}\left(x^{\prime }|x\right)/P_{X^{\prime }}\left(x^{\prime }\right) & if\;P_{X^{\prime }}\left(x^{\prime }\right)>0,\\ 1/|X| & otherwise, \end{array}\right. \end{array}$$

(121) $$\begin{array}{cc} P_{Y|X^{\prime }}\left(y|x^{\prime }\right) & ≜\sum_{x}B_{X|X^{\prime }}\left(x|x^{\prime }\right)\;P_{Y|X}\left(y|x\right). \end{array}$$

*Then, for all* α∈[0,∞] *,*

(122) $$\begin{array}{c} D_{\alpha }^{l}(P_{Y|X^{\prime }}\|Q_{Y}|P_{X^{\prime }})\leq D_{\alpha }^{l}(P_{Y|X}\|Q_{Y}\left|P_{X}\right). \end{array}$$

Once we provide the operational meaning of $D_{\alpha }^{l}\left(\cdot \right)$ in horse betting with side information (Theorem 10 below), Theorem 6 will become very intuitive: it expresses the fact that preprocessing the side information cannot increase the gambler’s utility; see Remark 8. Note that $P_{X^{\prime }}$ and $P_{Y|X^{\prime }}$ in Theorem 6 can be obtained from the following marginalization:

(123) $$\begin{array}{cc} P_{X^{\prime }}\left(x^{\prime }\right)\;P_{Y|X^{\prime }}\left(y|x^{\prime }\right) & =\sum_{x}P_{X}\left(x\right)\;B_{X^{\prime }|X}\left(x^{\prime }|x\right)\;P_{Y|X}\left(y|x\right). \end{array}$$

**Proof** **of** **Theorem** **6.** We show (122) for α∈(0,1)∪(1,∞); the claim will then extend to α∈[0,∞] by the continuity of $D_{\alpha }^{l}\left(\cdot \right)$ in α (Proposition 6 (c)). Consider first α∈(1,∞). Then, (122) holds because

$$\begin{array}{cc} \; & \frac{\alpha -1}{\alpha }\;D_{\alpha }^{l}(P_{Y|X^{\prime }}\|Q_{Y}\left|P_{X^{\prime }}\right) \end{array}$$

(124) $$\begin{array}{cc} \; & \;=\log\sum_{x^{\prime }\in \mathrm{supp}\left(P_{X^{\prime }}\right)}P_{X^{\prime }}\left(x^{\prime }\right){\left[\sum_{y}P_{Y|X^{\prime }}{\left(y|x^{\prime }\right)}^{\alpha }\;Q_{Y}{\left(y\right)}^{1-\alpha }\right]}^{\frac{1}{\alpha }} \end{array}$$

(125) $$\begin{array}{cc} \; & \;=\log\sum_{x^{\prime }\in \mathrm{supp}\left(P_{X^{\prime }}\right)}P_{X^{\prime }}\left(x^{\prime }\right){\left[\sum_{y}{\left[\sum_{x}B_{X|X^{\prime }}\left(x|x^{\prime }\right)\;P_{Y|X}\left(y|x\right)\;Q_{Y}{\left(y\right)}^{\frac{1-\alpha }{\alpha }}\right]}^{\alpha }\right]}^{\frac{1}{\alpha }} \end{array}$$

(126) $$\begin{array}{cc} \; & \;=\log\sum_{x^{\prime }\in \mathrm{supp}\left(P_{X^{\prime }}\right)}{\left[\sum_{y}{\left[\;\sum_{x\in \mathrm{supp}(P_{X})}P_{X}\left(x\right)\;B_{X^{\prime }|X}\left(x^{\prime }|x\right)\;P_{Y|X}\left(y|x\right)\;Q_{Y}{\left(y\right)}^{\frac{1-\alpha }{\alpha }}\right]}^{\alpha }\;\right]}^{\frac{1}{\alpha }} \end{array}$$

(127) $$\begin{array}{cc} \; & \;\leq \log\sum_{x^{\prime }\in \mathrm{supp}\left(P_{X^{\prime }}\right)}\sum_{x\in \mathrm{supp}(P_{X})}{\left[\sum_{y}{\left[P_{X}\left(x\right)\;B_{X^{\prime }|X}\left(x^{\prime }|x\right)\;P_{Y|X}\left(y|x\right)\;Q_{Y}{\left(y\right)}^{\frac{1-\alpha }{\alpha }}\right]}^{\alpha }\right]}^{\frac{1}{\alpha }} \end{array}$$

(128) $$\begin{array}{cc} \; & \;=\log\sum_{x\in \mathrm{supp}(P_{X})}P_{X}\left(x\right)\;\left[\;\sum_{x^{\prime }\in \mathrm{supp}\left(P_{X^{\prime }}\right)}B_{X^{\prime }|X}\left(x^{\prime }|x\right)\right]{\left[\sum_{y}P_{Y|X}{\left(y|x\right)}^{\alpha }\;Q_{Y}{\left(y\right)}^{1-\alpha }\right]}^{\frac{1}{\alpha }} \end{array}$$

(129) $$\begin{array}{cc} \; & \;=\log\sum_{x\in \mathrm{supp}(P_{X})}P_{X}\left(x\right)\;{\left[\sum_{y}P_{Y|X}{\left(y|x\right)}^{\alpha }\;Q_{Y}{\left(y\right)}^{1-\alpha }\right]}^{\frac{1}{\alpha }} \end{array}$$

(130) $$\begin{array}{cc} \; & \;=\frac{\alpha -1}{\alpha }\;D_{\alpha }^{l}(P_{Y|X}\|Q_{Y}\left|P_{X}\right), \end{array}$$

where (124) follows from (78); (125) follows from (121); (126) follows from (120); (127) follows from the Minkowski inequality [16] (III 2.4 Theorem 9); (129) holds because $P_{X}\left(x\right)>0$ and $P_{X^{\prime }}\left(x^{\prime }\right)=0$ imply $B_{X^{\prime }|X}\left(x^{\prime }|x\right)=0$, hence the first expression in square brackets on the left-hand side (LHS) of (129) equals one; and (130) follows from (78).

The proof for α∈(0,1) is very similar: (124)–(126) and (128)–(130) continue to hold, and (127) is reversed [16] (III 2.4 Theorem 9). Because now $\frac{\alpha -1}{\alpha }<0$, (122) continues to hold for α∈(0,1). □

As a special case of Theorem 6, we obtain the following relation between the conditional and the unconditional Rényi divergence:

**Corollary** **3.** *Let* $P_{X}$ *and* $Q_{Y}$ *be PMFs, and let* $P_{Y|X}$ *be a conditional PMF. Define the marginal PMF*

(131) $$\begin{array}{cc} P_{Y}\left(y\right) & ≜\sum_{x}P_{X}\left(x\right)\;P_{Y|X}\left(y|x\right). \end{array}$$

*Then, for all* α∈[0,∞] *,*

(132) $$\begin{array}{c} D_{\alpha }\left(P_{Y}\|Q_{Y}\right)\leq D_{\alpha }^{l}(P_{Y|X}\|Q_{Y}\left|P_{X}\right). \end{array}$$

**Proof.**  This follows from Theorem 6 in the same way that Corollary 1 followed from Theorem 2. □

Consider next $Q_{Y|X}$ that does depend on *X*. It turns out that Corollary 3, and hence Theorem 6, cannot be extended to this setting:

**Example** **2.** *Let* X={0,1} *and* Y={0,1,2} *. Define the PMFs* $P_{X}$ *,* $P_{Y|X}$ *, and* $Q_{Y|X}$ *as*

(133) $$\begin{array}{cccc} P_{X}\left(0\right) & =0.5, & P_{X}\left(1\right) & =0.5, \end{array}$$

(134) $$\begin{array}{cccccc} P_{Y|X}\left(0|0\right) & =0.96, & \;P_{Y|X}\left(1|0\right) & =0.02, & \;P_{Y|X}\left(2|0\right) & =0.02, \end{array}$$

(135) $$\begin{array}{cccccc} P_{Y|X}\left(0|1\right) & =0.12, & \;P_{Y|X}\left(1|1\right) & =0.02, & \;P_{Y|X}\left(2|1\right) & =0.86, \end{array}$$

(136) $$\begin{array}{cccccc} Q_{Y|X}\left(0|0\right) & =0.06, & \;Q_{Y|X}\left(1|0\right) & =0.92, & \;Q_{Y|X}\left(2|0\right) & =0.02, \end{array}$$

(137) $$\begin{array}{cccccc} Q_{Y|X}\left(0|1\right) & =0.02, & \;Q_{Y|X}\left(1|1\right) & =0.16, & \;Q_{Y|X}\left(2|1\right) & =0.82. \end{array}$$

*Then, for* α=0.5 *and for* α=2 *,*

(138) $$D_{\alpha }(P_{Y}\|Q_{Y})>D_{\alpha }^{l}(P_{Y|X}\|Q_{Y|X}\left|P_{X}\right),$$

*where the PMFs* $P_{Y}$ *and* $Q_{Y}$ *are given by*

(139) $$\begin{array}{cc} P_{Y}\left(y\right) & ≜\sum_{x}P_{X}\left(x\right)\;P_{Y|X}\left(y|x\right), \end{array}$$

(140) $$\begin{array}{cc} Q_{Y}\left(y\right) & ≜\sum_{x}P_{X}\left(x\right)\;Q_{Y|X}\left(y|x\right). \end{array}$$

**Proof.**  Numerically, $D_{0.5}\left(P_{Y}\|Q_{Y}\right)\approx 1.11$ bits, which is larger than $D_{0.5}^{l}(P_{Y|X}\|Q_{Y|X}\left|P_{X}\right)\approx 0.93$ bits. Similarly, $D_{2}\left(P_{Y}\|Q_{Y}\right)\approx 2.95$ bits, which is larger than $D_{2}^{l}(P_{Y|X}\|Q_{Y|X}\left|P_{X}\right)\approx 2.75$ bits. □

## 6. Relation to Arimoto’s Measures

Before discussing Arimoto’s measures, we first recall the definition of the Rényi entropy. The Rényi entropy of order α [7] is defined for all positive α’s other than one as

(141) $$\begin{array}{c} H_{\alpha }\left(X\right)≜\frac{1}{1-\alpha }\log\sum_{x}P{\left(x\right)}^{\alpha }. \end{array}$$

For α being zero, one, or infinity, we define by continuous extension of (141)

(142) $$\begin{array}{cc} H_{0}\left(X\right) & ≜\log\;|\mathrm{supp}\left(P_{X}\right)|, \end{array}$$

(143) $$\begin{array}{cc} H_{1}\left(X\right) & ≜H(X), \end{array}$$

(144) $$\begin{array}{cc} H_{\infty }\left(X\right) & ≜-\log\max_{x}P\left(x\right), \end{array}$$

where H(X) denotes Shannon’s entropy. The Rényi entropy can be related to the Rényi divergence as follows:

(145) $$\begin{array}{c} H_{\alpha }\left(X\right)=\log\;\left|X\right|-D_{\alpha }\left(P_{X}\|U_{X}\right), \end{array}$$

where $U_{X}$ denotes the uniform distribution over X.

There are different ways to define a conditional Rényi entropy [40]; we use Arimoto’s proposal. The Arimoto–Rényi conditional entropy of order α [12,38,40,41] is defined for positive α other than one as

(146) $$\begin{array}{cc} H_{\alpha }\left(X|Y\right) & ≜\frac{\alpha }{1-\alpha }\log\sum_{y\in \mathrm{supp}(P_{Y})}P\left(y\right){\left[\sum_{x}P{\left(x|y\right)}^{\alpha }\right]}^{\frac{1}{\alpha }} \end{array}$$

(147) $$\begin{array}{cc} \; & =\frac{\alpha }{1-\alpha }\log\sum_{y\in \mathrm{supp}(P_{Y})}P\left(y\right)\;2^{\frac{1-\alpha }{\alpha }H_{\alpha }\left(P_{X|Y=y}\right)}, \end{array}$$

where (147) follows from the definition of the Rényi entropy in (141). The Arimoto–Rényi conditional entropy plays a key role in guessing with side information [20,42,43,44] and in task encoding with side information [45]; and it can be related to hypothesis testing [41]. For α being zero, one, or infinity, we define by continuous extension of (146)

(148) $$\begin{array}{cc} H_{0}\left(X|Y\right) & ≜\log\max_{y\in \mathrm{supp}(P_{Y})}\left|\mathrm{supp}(P_{X|Y=y})\right|, \end{array}$$

(149) $$\begin{array}{cc} H_{1}\left(X|Y\right) & ≜H(X|Y), \end{array}$$

(150) $$\begin{array}{cc} H_{\infty }\left(X|Y\right) & ≜-\log\sum_{y\in \mathrm{supp}(P_{Y})}P\left(y\right)\max_{x}P\left(x|y\right), \end{array}$$

where H(X|Y) denotes Shannon’s conditional entropy. The analog of (145) for $H_{\alpha }\left(X|Y\right)$ is:

**Remark** **2.** *For all* α∈[0,∞] *,*

(151) $$\begin{array}{cc} H_{\alpha }\left(X|Y\right) & =\log\;\left|X\right|-D_{\alpha }^{l}(P_{X|Y}\|U_{X}\left|P_{Y}\right) \end{array}$$

(152) $$\begin{array}{cc} \; & =\log\;\left|X\right|-\min_{Q_{Y}}D_{\alpha }\left(P_{Y}P_{X|Y}\|Q_{Y}U_{X}\right). \end{array}$$

**Proof.**  Equation (151) follows, using some algebra, from the definition of $D_{\alpha }^{l}\left(\cdot \right)$ in (78)–(81); and (152) follows from Proposition 4. (The characterization in (152) previously appeared as [40] (Theorem 4).) □

Arimoto [12] also defined the following measure of dependence:

(153) $$\begin{array}{cc} I_{\alpha }^{a}\left(X;Y\right) & ≜H_{\alpha }\left(X\right)-H_{\alpha }\left(X|Y\right) \end{array}$$

(154) $$\begin{array}{cc} \; & =\frac{\alpha }{\alpha -1}\log\sum_{y}{\left[\sum_{x}\frac{P{\left(x\right)}^{\alpha }}{\sum_{x^{\prime }\in X}P{\left(x^{\prime }\right)}^{\alpha }}\;P{\left(y|x\right)}^{\alpha }\right]}^{\frac{1}{\alpha }}, \end{array}$$

where (154) follows from (141) and (146). Using Remark 2, we can express $I_{\alpha }^{a}\left(X;Y\right)$ in terms of $D_{\alpha }^{l}\left(\cdot \right)$:

**Remark** **3.** *For all* α∈[0,∞] *,*

(155) $$\begin{array}{c} I_{\alpha }^{a}\left(X;Y\right)=D_{\alpha }^{l}(P_{X|Y}\|U_{X}\left|P_{Y}\right)-D_{\alpha }\left(P_{X}\|U_{X}\right). \end{array}$$

**Proof.**  This follows from (145), (151), and (153). □

## 7. Relations Between the Conditional Rényi Divergences and the Rényi Dependence Measures

In this section, we first establish the greater-or-equal-than order between the conditional Rényi divergences, where the order depends on whether α∈[0,1] or α∈[1,∞]. We then show that this implies the same order between the dependence measures derived from the conditional Rényi divergences. Finally, we remark that many of the dependence measures coincide when they are maximized over all PMFs $P_{X}$.

**Proposition** **7.** *For all* α∈[0,∞] *,*

(156) $$\begin{array}{c} D_{\alpha }^{l}(P_{Y|X}\|Q_{Y|X}|P_{X})\leq D_{\alpha }^{s}(P_{Y|X}\|Q_{Y|X}\left|P_{X}\right). \end{array}$$

**Proof.**  This holds because

(157) $$\begin{array}{cc} D_{\alpha }^{l}(P_{Y|X}\|Q_{Y|X}\left|P_{X}\right) & =\min_{Q_{X}}D_{\alpha }\left(P_{X}P_{Y|X}\|Q_{X}Q_{Y|X}\right) \end{array}$$

(158) $$\begin{array}{cc} \; & \leq D_{\alpha }\left(P_{X}P_{Y|X}\|P_{X}Q_{Y|X}\right) \end{array}$$

(159) $$\begin{array}{cc} \; & =D_{\alpha }^{s}(P_{Y|X}\|Q_{Y|X}\left|P_{X}\right), \end{array}$$

where (157) follows from Proposition 4, and (159) follows from the definition of $D_{\alpha }^{s}\left(\cdot \right)$ in (54). □

**Theorem** **7.** *For all* α∈[0,1] *,*

(160) $$\begin{array}{c} D_{\alpha }^{l}(P_{Y|X}\|Q_{Y|X}|P_{X})\leq D_{\alpha }^{s}(P_{Y|X}\|Q_{Y|X}|P_{X})\leq D_{\alpha }^{c}(P_{Y|X}\|Q_{Y|X}\left|P_{X}\right). \end{array}$$

*For all* α∈[1,∞] *,*

(161) $$\begin{array}{c} D_{\alpha }^{c}(P_{Y|X}\|Q_{Y|X}|P_{X})\leq D_{\alpha }^{l}(P_{Y|X}\|Q_{Y|X}|P_{X})\leq D_{\alpha }^{s}(P_{Y|X}\|Q_{Y|X}\left|P_{X}\right). \end{array}$$

**Proof.**  For both α∈[0,1] and α∈[1,∞], the relation $D_{\alpha }^{l}\left(\cdot \right)\leq D_{\alpha }^{s}\left(\cdot \right)$ follows from Proposition 7.

We next show that $D_{\alpha }^{s}\left(\cdot \right)\leq D_{\alpha }^{c}\left(\cdot \right)$ for α∈[0,1]. We show this for α∈(0,1); the claim will then extend to α∈[0,1] by the continuity in α of $D_{\alpha }^{s}\left(\cdot \right)$ and $D_{\alpha }^{c}\left(\cdot \right)$ (Proposition 3 (c) and Proposition 2 (c)). For α∈(0,1),

(162) $$\begin{array}{cc} \left(\alpha -1\right)\;D_{\alpha }^{s}(P_{Y|X}\|Q_{Y|X}\left|P_{X}\right) & =\log\sum_{x\in \mathrm{supp}(P_{X})}P\left(x\right)\sum_{y}P{\left(y|x\right)}^{\alpha }\;Q{\left(y|x\right)}^{1-\alpha } \end{array}$$

(163) $$\begin{array}{cc} \; & \geq \sum_{x\in \mathrm{supp}(P_{X})}P\left(x\right)\log\sum_{y}P{\left(y|x\right)}^{\alpha }\;Q{\left(y|x\right)}^{1-\alpha } \end{array}$$

(164) $$\begin{array}{cc} \; & =\left(\alpha -1\right)\;D_{\alpha }^{c}(P_{Y|X}\|Q_{Y|X}\left|P_{X}\right), \end{array}$$

where (162) follows from (55); (163) follows from Jensen’s inequality because log(·) is a concave function; and (164) follows from (30). The proof of the claim for α∈(0,1) is finished by dividing (162)–(164) by α−1, which reverses the inequality because α−1<0.

We conclude by showing that $D_{\alpha }^{c}\left(\cdot \right)\leq D_{\alpha }^{l}\left(\cdot \right)$ for α∈[1,∞]. We show this for α∈(1,∞); the claim will then extend to α∈[1,∞] by the continuity of $D_{\alpha }^{c}\left(\cdot \right)$ and $D_{\alpha }^{l}\left(\cdot \right)$ in α (Proposition 2 (c) and Proposition 6 (c)). For α∈(1,∞),

(165) $$\begin{array}{cc} D_{\alpha }^{c}(P_{Y|X}\|Q_{Y|X}\left|P_{X}\right) & =\sum_{x\in \mathrm{supp}(P_{X})}P\left(x\right)\;\frac{1}{\alpha -1}\log\sum_{y}P{\left(y|x\right)}^{\alpha }\;Q{\left(y|x\right)}^{1-\alpha } \end{array}$$

(166) $$\begin{array}{cc} \; & =\frac{\alpha }{\alpha -1}\sum_{x\in \mathrm{supp}(P_{X})}P\left(x\right)\log{\left[\sum_{y}P{\left(y|x\right)}^{\alpha }\;Q{\left(y|x\right)}^{1-\alpha }\right]}^{\frac{1}{\alpha }} \end{array}$$

(167) $$\begin{array}{cc} \; & \leq \frac{\alpha }{\alpha -1}\log\sum_{x\in \mathrm{supp}(P_{X})}P\left(x\right)\;{\left[\sum_{y}P{\left(y|x\right)}^{\alpha }\;Q{\left(y|x\right)}^{1-\alpha }\right]}^{\frac{1}{\alpha }} \end{array}$$

(168) $$\begin{array}{cc} \; & =D_{\alpha }^{l}(P_{Y|X}\|Q_{Y|X}\left|P_{X}\right), \end{array}$$

where (165) follows from (30); (167) follows from Jensen’s inequality because log(·) is a concave function; and (168) follows from (78). □

**Corollary** **4.** *For all* α∈[0,1] *,*

(169) $$\begin{array}{c} J_{\alpha }\left(X;Y\right)\leq I_{\alpha }^{s}\left(X;Y\right)\leq I_{\alpha }^{c}\left(X;Y\right). \end{array}$$

*For all* α∈[1,∞] *,*

(170) $$\begin{array}{c} I_{\alpha }^{c}\left(X;Y\right)\leq J_{\alpha }\left(X;Y\right)\leq I_{\alpha }^{s}\left(X;Y\right). \end{array}$$

**Proof.**  By (34) and (60) and Proposition 5, respectively,

(171) $$\begin{array}{cc} I_{\alpha }^{c}\left(X;Y\right) & =\min_{Q_{Y}}D_{\alpha }^{c}(P_{Y|X}\|Q_{Y}\left|P_{X}\right), \end{array}$$

(172) $$\begin{array}{cc} I_{\alpha }^{s}\left(X;Y\right) & =\min_{Q_{Y}}D_{\alpha }^{s}(P_{Y|X}\|Q_{Y}\left|P_{X}\right), \end{array}$$

(173) $$\begin{array}{cc} J_{\alpha }\left(X;Y\right) & =\min_{Q_{Y}}D_{\alpha }^{l}(P_{Y|X}\|Q_{Y}\left|P_{X}\right). \end{array}$$

The corollary now follows from (171)–(173) and Theorem 7. □

Despite $I_{\alpha }^{c}\left(X;Y\right)$, $I_{\alpha }^{s}\left(X;Y\right)$, $I_{\alpha }^{a}\left(X;Y\right)$, and $J_{\alpha }\left(X;Y\right)$ being different measures, they often coincide when maximized over all PMFs $P_{X}$:

**Theorem** **8.** *For every conditional PMF* $P_{Y|X}$ *and every* α∈(0,1)∪(1,∞) *,*

(174) $$\begin{array}{cc} \max_{P_{X}}I_{\alpha }^{c}\left(P_{X},P_{Y|X}\right) & =\max_{P_{X}}I_{\alpha }^{s}\left(P_{X},P_{Y|X}\right) \end{array}$$

(175) $$\begin{array}{cc} \; & =\max_{P_{X}}I_{\alpha }^{a}\left(P_{X},P_{Y|X}\right). \end{array}$$

*In addition, for every conditional PMF* $P_{Y|X}$ *and every* $\alpha \in \left[\frac{1}{2},1\right)\cup \left(1,\infty \right)$ *,*

(176) $$\begin{array}{c} \max_{P_{X}}J_{\alpha }\left(P_{X},P_{Y|X}\right)=\max_{P_{X}}I_{\alpha }^{s}\left(P_{X},P_{Y|X}\right). \end{array}$$

*For* $\alpha \in (0,\frac{1}{2})$ *, the situation is different: there exists a conditional PMF* $P_{Y|X}$ *such that, for every* $\alpha \in (0,\frac{1}{2})$ *,*

(177) $$\begin{array}{c} \max_{P_{X}}J_{\alpha }\left(P_{X},P_{Y|X}\right)<\max_{P_{X}}I_{\alpha }^{s}\left(P_{X},P_{Y|X}\right). \end{array}$$

**Proof.**  Equation (174) follows from [9] (Proposition 1); (175) follows from [12] (Lemma 1); and (176) follows from [38] (Theorem V.1) for α∈(1,∞).

We next establish (176) for $\alpha \in [\frac{1}{2},1)$. Observe that, for $\alpha \in [\frac{1}{2},1)$, (176) is equivalent to

(178) $$\begin{array}{cc} \max_{P_{X}}-2^{\frac{\alpha -1}{\alpha }J_{\alpha }\left(P_{X},P_{Y|X}\right)} & =\max_{P_{X}}-2^{\frac{\alpha -1}{\alpha }I_{\alpha }^{s}\left(P_{X},P_{Y|X}\right)}. \end{array}$$

For $\alpha \in [\frac{1}{2},1)$, (178) holds because

(179) $$\begin{array}{cc} \max_{P_{X}}-2^{\frac{\alpha -1}{\alpha }J_{\alpha }\left(P_{X},P_{Y|X}\right)} & =\max_{P_{X}}\min_{Q_{Y}}-2^{\frac{\alpha -1}{\alpha }D_{\alpha }^{l}(P_{Y|X}\|Q_{Y}\left|P_{X}\right)} \end{array}$$

(180) $$\begin{array}{cc} \; & =-\min_{P_{X}}\max_{Q_{Y}}\sum_{x}P_{X}\left(x\right)\;{\left[\sum_{y}P{\left(y|x\right)}^{\alpha }\;Q_{Y}{\left(y\right)}^{1-\alpha }\right]}^{\frac{1}{\alpha }} \end{array}$$

(181) $$\begin{array}{cc} \; & =-\max_{Q_{Y}}\min_{P_{X}}\sum_{x}P_{X}\left(x\right)\;{\left[\sum_{y}P{\left(y|x\right)}^{\alpha }\;Q_{Y}{\left(y\right)}^{1-\alpha }\right]}^{\frac{1}{\alpha }} \end{array}$$

(182) $$\begin{array}{cc} \; & =-\max_{Q_{Y}}\min_{x}{\left[\sum_{y}P{\left(y|x\right)}^{\alpha }\;Q_{Y}{\left(y\right)}^{1-\alpha }\right]}^{\frac{1}{\alpha }} \end{array}$$

(183) $$\begin{array}{cc} \; & =-{\left[\max_{Q_{Y}}\min_{x}\sum_{y}P{\left(y|x\right)}^{\alpha }\;Q_{Y}{\left(y\right)}^{1-\alpha }\right]}^{\frac{1}{\alpha }} \end{array}$$

(184) $$\begin{array}{cc} \; & =-{\left[\max_{Q_{Y}}\min_{P_{X}}\sum_{x}P_{X}\left(x\right)\sum_{y}P{\left(y|x\right)}^{\alpha }\;Q_{Y}{\left(y\right)}^{1-\alpha }\right]}^{\frac{1}{\alpha }} \end{array}$$

(185) $$\begin{array}{cc} \; & =-{\left[\min_{P_{X}}\max_{Q_{Y}}\sum_{x}P_{X}\left(x\right)\sum_{y}P{\left(y|x\right)}^{\alpha }\;Q_{Y}{\left(y\right)}^{1-\alpha }\right]}^{\frac{1}{\alpha }} \end{array}$$

(186) $$\begin{array}{cc} \; & =-\min_{P_{X}}\max_{Q_{Y}}{\left[\sum_{x}P_{X}\left(x\right)\sum_{y}P{\left(y|x\right)}^{\alpha }\;Q_{Y}{\left(y\right)}^{1-\alpha }\right]}^{\frac{1}{\alpha }} \end{array}$$

(187) $$\begin{array}{cc} \; & =\max_{P_{X}}\min_{Q_{Y}}-2^{\frac{\alpha -1}{\alpha }D_{\alpha }^{s}(P_{Y|X}\|Q_{Y}\left|P_{X}\right)} \end{array}$$

(188) $$\begin{array}{cc} \; & =\max_{P_{X}}-2^{\frac{\alpha -1}{\alpha }I_{\alpha }^{s}\left(P_{X},P_{Y|X}\right)}, \end{array}$$

where (179) follows from Proposition 5; (180) follows from (78); (181) and (185) follow from a minimax theorem and are justified below; (187) follows from (55); and (188) follows from (60).

To justify (181), we apply the minimax theorem [46] (Corollary 37.3.2) to the function f:P(Y)×P(X)→R,

(189) $$\begin{array}{c} f\left(Q_{Y},P_{X}\right)=\sum_{x}P_{X}\left(x\right)\;{\left[\sum_{y}P{\left(y|x\right)}^{\alpha }\;Q_{Y}{\left(y\right)}^{1-\alpha }\right]}^{\frac{1}{\alpha }}. \end{array}$$

The sets of all PMFs over X and over Y are convex and compact; the function *f* is jointly continuous in the pair $\left(Q_{Y},P_{X}\right)$ because it is a composition of continuous functions; for every $Q_{Y}\in P\left(Y\right)$, the function *f* is linear and hence convex in $P_{X}$; and it only remains to show that the function *f* is concave in $Q_{Y}$ for every $P_{X}\in P\left(X\right)$. Indeed, for every $\lambda ,\lambda^{\prime }\in \left[0,1\right]$ with $\lambda +\lambda^{\prime }=1$, every $Q_{Y},Q_{Y}^{\prime }\in P\left(Y\right)$, and every $P_{X}\in P\left(X\right)$,

(190) $$\begin{array}{cc} \; & f(\lambda \;Q_{Y}+\lambda^{\prime }\;Q_{Y}^{\prime },P_{X}) \end{array}$$

(191) $$\begin{array}{cc} \; & \;=\sum_{x}P_{X}\left(x\right)\;{\left[\sum_{y}P{\left(y|x\right)}^{\alpha }\;{\left[\lambda \;Q_{Y}\left(y\right)+\lambda^{\prime }\;Q_{Y}^{\prime }\left(y\right)\right]}^{1-\alpha }\right]}^{\frac{1}{\alpha }} \end{array}$$

(192) $$\begin{array}{cc} \; & \;=\sum_{x}P_{X}\left(x\right)\;{\left[\sum_{y}{\left[\lambda \;P{\left(y|x\right)}^{\frac{\alpha }{1-\alpha }}\;Q_{Y}\left(y\right)+\lambda^{\prime }\;P{\left(y|x\right)}^{\frac{\alpha }{1-\alpha }}\;Q_{Y}^{\prime }\left(y\right)\right]}^{1-\alpha }\right]}^{\frac{1}{1-\alpha }\cdot \frac{1-\alpha }{\alpha }} \end{array}$$

(193) $$\begin{array}{cc} \; & \;\geq \sum_{x}P_{X}\left(x\right)\;\{{\left[\sum_{y}{\left[\lambda \;P{\left(y|x\right)}^{\frac{\alpha }{1-\alpha }}\;Q_{Y}\left(y\right)\right]}^{1-\alpha }\right]}^{\frac{1}{1-\alpha }}+{\left[\sum_{y}{\left[\lambda^{\prime }\;P{\left(y|x\right)}^{\frac{\alpha }{1-\alpha }}\;Q_{Y}^{\prime }\left(y\right)\right]}^{1-\alpha }\right]}^{\frac{1}{1-\alpha }}{\}}^{\frac{1-\alpha }{\alpha }} \end{array}$$

(194) $$\begin{array}{cc} \; & \;=\sum_{x}P_{X}\left(x\right)\;\{\lambda \;{\left[\sum_{y}P{\left(y|x\right)}^{\alpha }\;Q_{Y}{\left(y\right)}^{1-\alpha }\right]}^{\frac{1}{1-\alpha }}+\lambda^{\prime }\;{\left[\sum_{y}P{\left(y|x\right)}^{\alpha }\;Q_{Y}^{\prime }{\left(y\right)}^{1-\alpha }\right]}^{\frac{1}{1-\alpha }}{\}}^{\frac{1-\alpha }{\alpha }} \end{array}$$

(195) $$\begin{array}{cc} \; & \;\geq \sum_{x}P_{X}\left(x\right)\;\{\lambda \;{\left[\sum_{y}P{\left(y|x\right)}^{\alpha }\;Q_{Y}{\left(y\right)}^{1-\alpha }\right]}^{\frac{1}{\alpha }}+\lambda^{\prime }\;{\left[\sum_{y}P{\left(y|x\right)}^{\alpha }\;Q_{Y}^{\prime }{\left(y\right)}^{1-\alpha }\right]}^{\frac{1}{\alpha }}\} \end{array}$$

(196) $$\begin{array}{cc} \; & \;=\lambda \;f\left(Q_{Y},P_{X}\right)+\lambda^{\prime }\;f\left(Q_{Y}^{\prime },P_{X}\right), \end{array}$$

where (193) follows from the reverse Minkowski inequality [16] (III 2.4 Theorem 9) because $\alpha \in [\frac{1}{2},1)$; and (195) holds because the function $z\mapsto z^{(1-\alpha )/\alpha }$ is concave for $\alpha \in [\frac{1}{2},1)$.

The justification of (185) is very similar to that of (181); here, we apply the minimax theorem to the function g:P(Y)×P(X)→R,

(197) $$\begin{array}{c} g\left(Q_{Y},P_{X}\right)=\sum_{x}P_{X}\left(x\right)\sum_{y}P{\left(y|x\right)}^{\alpha }\;Q_{Y}{\left(y\right)}^{1-\alpha }. \end{array}$$

Compared to the justification of (181), the only essential difference lies in showing that the function *g* is concave in $Q_{Y}$ for every $P_{X}\in P\left(X\right)$: here, this follows easily from the concavity of the function $z\mapsto z^{1-\alpha }$ for $\alpha \in [\frac{1}{2},1)$.

We conclude the proof by establishing (177). Let X=Y={0,1}, and let the conditional PMF $P_{Y|X}$ be given by $P_{Y|X}\left(y|x\right)=𝟙\left\{y=x\right\}$. (This corresponds to a binary noiseless channel.) Then, denoting by $U_{X}$ the uniform distribution over X,

(198) $$\begin{array}{cc} \max_{P_{X}}I_{\alpha }^{s}\left(P_{X},P_{Y|X}\right) & \geq I_{\alpha }^{s}\left(U_{X},P_{Y|X}\right) \end{array}$$

(199) $$\begin{array}{cc} \; & =\log2, \end{array}$$

where (199) follows from (61). On the other hand, for every $\alpha \in (0,\frac{1}{2})$ and every PMF $P_{X}$,

(200) $$\begin{array}{cc} J_{\alpha }\left(P_{X},P_{Y|X}\right) & =\frac{\alpha }{1-\alpha }\;H_{\infty }\left(P_{X}\right) \end{array}$$

(201) $$\begin{array}{cc} \; & \leq \frac{\alpha }{1-\alpha }\log2 \end{array}$$

(202) $$\begin{array}{cc} \; & <\log2, \end{array}$$

where (200) follows from [3] (Lemma 11); (201) follows from (144); and (202) holds because $\alpha \in (0,\frac{1}{2})$. Inequality (177) now follows from (199) and (202). □

## 8. Horse Betting

In this section, we analyze horse betting with a gambler investing all her money. Recall from the introduction that the winning horse *X* is distributed according to the PMF *p*, where we assume p(x)>0 for all x∈X; that the odds offered by the bookmaker are denoted by o:X→(0,∞); that the fraction of her wealth that the gambler bets on Horse x∈X is denoted b(x)≥0; that the wealth relative is the random variable S≜b(X)o(X); and that we seek betting strategies that maximize the utility function

(203) $$\begin{array}{c} U_{\beta }≜\left\{\begin{array}{cc} \frac{1}{\beta }\log E\left[S^{\beta }\right] & \mathrm{if}\;\beta \neq 0,\\ E[\log S] & \mathrm{if}\;\beta =0. \end{array}\right. \end{array}$$

Because the gambler invests all her money, *b* is a PMF. As in [47] (Section 10.3), define the constant

(204) $$\begin{array}{c} c≜{\left[\sum_{x}\frac{1}{o(x)}\right]}^{-1} \end{array}$$

and the PMF

(205) $$\begin{array}{c} r\left(x\right)≜\frac{c}{o(x)}. \end{array}$$

Using these definitions, the utility function $U_{\beta }$ can be decomposed as follows:

**Theorem** **9.** *Let* β∈(−∞,1) *, and let b be a PMF. Then,*

(206) $$\begin{array}{c} U_{\beta }=\log c+D_{\;\frac{1}{1-\beta }\;}\left(p\|r\right)-D_{1-\beta }\left(g^{\left(\beta \right)}\|b\right), \end{array}$$

*where the PMF* $g^{\left(\beta \right)}$ *is given by*

(207) $$\begin{array}{c} g^{\left(\beta \right)}\left(x\right)≜\frac{p{\left(x\right)}^{\frac{1}{1-\beta }}\;o{\left(x\right)}^{\frac{\beta }{1-\beta }}}{\sum_{x^{\prime }\in X}p{\left(x^{\prime }\right)}^{\frac{1}{1-\beta }}\;o{\left(x^{\prime }\right)}^{\frac{\beta }{1-\beta }}}. \end{array}$$

*Thus, choosing* $b=g^{\left(\beta \right)}$ *uniquely maximizes* $U_{\beta }$ *among all PMFs b.*

The three terms in (206) can be interpreted as follows:

1. The first term, logc, depends only on the odds and is related to the fairness of the odds. The odds are called subfair if c<1, fair if c=1, and superfair if c>1.
2. The second term, $D_{1/(1-\beta )}\left(p\|r\right)$, is related to the bookmaker’s estimate of the winning probabilities. It is zero if and only if the odds are inversely proportional to the winning probabilities.
3. The third term, $-D_{1-\beta }\left(g^{\left(\beta \right)}\|b\right)$, is related to the gambler’s estimate of the winning probabilities. It is zero if and only if *b* is equal to $g^{\left(\beta \right)}$.

**Remark** **4.** *For*β=0*,* (206) *reduces to the following decomposition of the doubling rate* E[logS]*:*

(208) $$\begin{array}{c} E[\log S]=\log c+D(p\|r)-D(p\|b). \end{array}$$

*(This decomposition appeared previously in [47] (Section 10.3).) Equation* (208) *implies that the doubling rate is maximized by proportional gambling, i.e., that* E[logS] *is maximized if and only if b is equal to p.*

**Remark** **5.** *Considering the limits*β→−∞*and*β↑1*, the PMF*$g^{\left(\beta \right)}$*satisfies, for every*x∈X*,*

(209) $$\begin{array}{cc} \lim_{\beta \to -\infty }g^{\left(\beta \right)}\left(x\right) & =\frac{c}{o(x)}, \end{array}$$

(210) $$\begin{array}{cc} \lim_{\beta ↑1}g^{\left(\beta \right)}\left(x\right) & =\frac{p(x)\;𝟙\{x\in S\}}{\sum_{x^{\prime }\in X}p\left(x^{\prime }\right)\;𝟙\left\{x^{\prime }\in S\right\}}, \end{array}$$

*where the set*S*is defined as*$S≜\left\{x^{\prime }\in X:p\left(x^{\prime }\right)\;o\left(x^{\prime }\right)=\max_{x}\left[p\left(x\right)\;o\left(x\right)\right]\right\}$*. It follows from Proposition 8 below that the RHS of* (209) *is the unique maximizer of* $\lim_{\beta \to -\infty }U_{\beta }$*; and it follows from the proof of Proposition 9 below that the RHS of* (210) *is a maximizer (not necessarily unique) of* $U_{1}$*.*

**Proof** **of** **Remark** **5.** Recall that we assume p(x)>0 for every x∈X. Then, (209) follows from (207) and the definition of *c* in (204). To establish (210), define $\tau ≜\max_{x}\left[p\left(x\right)\;o\left(x\right)\right]$ and observe that, for every x∈X,

(211) $$\begin{array}{cc} \lim_{\beta ↑1}g^{\left(\beta \right)}\left(x\right) & =\lim_{\beta ↑1}\frac{p\left(x\right)\;{\left[p(x)\;o(x)/\tau \right]}^{\frac{\beta }{1-\beta }}}{\sum_{x^{\prime }\in X}p\left(x^{\prime }\right)\;{\left[p\left(x^{\prime }\right)\;o\left(x^{\prime }\right)/\tau \right]}^{\frac{\beta }{1-\beta }}} \end{array}$$

(212) $$\begin{array}{cc} \; & =\frac{p(x)\;𝟙\{x\in S\}}{\sum_{x^{\prime }\in X}p\left(x^{\prime }\right)\;𝟙\left\{x^{\prime }\in S\right\}}, \end{array}$$

where (211) follows from (207) and some algebra; and (212) is justified as follows: if x∈S, then ${\left[p(x)\;o(x)/\tau \right]}^{\beta /(1-\beta )}$ equals one; and if x∉S, then ${\left[p(x)\;o(x)/\tau \right]}^{\beta /(1-\beta )}$ tends to zero as β↑1 because p(x)o(x)/τ<1 and because $\lim_{\beta ↑1}\frac{\beta }{1-\beta }=+\infty$. □

**Remark** **6.** *Using the definition in* (24) *for the Rényi divergence of negative orders, it is not difficult to see from the proof of Theorem 9 below that* (206) *also holds for* β>1*. However, because the Rényi divergence of negative orders is nonpositive instead of nonnegative, the above interpretation is not valid anymore; in particular, for* β>1*, choosing* $b=g^{\left(\beta \right)}$ *is in general not optimal.*

**Proof** **of** **Theorem** **9.** We first show the maximization claim. The only term on the RHS of (206) that depends on *b* is $-D_{1-\beta }\left(g^{\left(\beta \right)}\|b\right)$. Because 1−β>0, this term is maximized if and only if $b=g^{\left(\beta \right)}$ (Proposition 1 (a)).

We now establish (206) for β∈(−∞,0)∪(0,1); we omit the proof for β=0, which can be found in [47] (Section 10.3). For β∈(−∞,0)∪(0,1),

(213) $$\begin{array}{c} U_{\beta }=\frac{1}{\beta }\log\sum_{x}p\left(x\right)\;b{\left(x\right)}^{\beta }\;o{\left(x\right)}^{\beta }. \end{array}$$

For every x∈X,

(214) $$\begin{array}{cc} p\left(x\right)\;b{\left(x\right)}^{\beta }\;o{\left(x\right)}^{\beta } & ={\left[\;\sum_{x^{\prime }\in X}p{\left(x^{\prime }\right)}^{\frac{1}{1-\beta }}\;o{\left(x^{\prime }\right)}^{\frac{\beta }{1-\beta }}\right]}^{1-\beta }\cdot g^{\left(\beta \right)}{\left(x\right)}^{1-\beta }\;b{\left(x\right)}^{\beta }, \end{array}$$

which follows from (207). Now, (206) holds because

(215) $$\begin{array}{cc} U_{\beta } & =\frac{1-\beta }{\beta }\log\sum_{x^{\prime }\in X}p{\left(x^{\prime }\right)}^{\frac{1}{1-\beta }}\;o{\left(x^{\prime }\right)}^{\frac{\beta }{1-\beta }}+\frac{1}{\beta }\log\sum_{x}g^{\left(\beta \right)}{\left(x\right)}^{1-\beta }\;b{\left(x\right)}^{\beta } \end{array}$$

(216) $$\begin{array}{cc} \; & =\frac{1-\beta }{\beta }\log\sum_{x^{\prime }\in X}p{\left(x^{\prime }\right)}^{\frac{1}{1-\beta }}\;o{\left(x^{\prime }\right)}^{\frac{\beta }{1-\beta }}-D_{1-\beta }\left(g^{\left(\beta \right)}\|b\right) \end{array}$$

(217) $$\begin{array}{cc} \; & =\log c+\frac{1-\beta }{\beta }\log\sum_{x^{\prime }\in X}p{\left(x^{\prime }\right)}^{\frac{1}{1-\beta }}\;r{\left(x^{\prime }\right)}^{\frac{-\beta }{1-\beta }}-D_{1-\beta }\left(g^{\left(\beta \right)}\|b\right) \end{array}$$

(218) $$\begin{array}{cc} \; & =\log c+D_{\;\frac{1}{1-\beta }\;}\left(p\|r\right)-D_{1-\beta }\left(g^{\left(\beta \right)}\|b\right), \end{array}$$

where (215) follows from (213) and (214); (216) follows from identifying the Rényi divergence (recall that $g^{\left(\beta \right)}$ and *b* are PMFs); (217) follows from (205); and (218) follows from identifying the Rényi divergence (recall that *r* is a PMF). □

The rest of the section presents the cases β→−∞, β≥1, and β→+∞.

**Proposition** **8.** *Let b be a PMF. Then,*

(219) $$\begin{array}{cc} \lim_{\beta \to -\infty }U_{\beta } & =\log\min_{x}\left[b(x)\;o(x)\right] \end{array}$$

(220) $$\begin{array}{cc} \; & \leq \log c. \end{array}$$

*Inequality* (220) *holds with equality if and only if* b(x)=c/o(x) *for all* x∈X*.*

Observe that if b(x)=c/o(x) for all x∈X, then S=c with probability one, i.e., *S* does not depend on the winning horse.

**Proof** **of** **Proposition** **8.** Equation (219) holds because

(221) $$\begin{array}{cc} \lim_{\beta \to -\infty }U_{\beta } & =\lim_{\beta \to -\infty }\log{\left[\sum_{x}p\left(x\right)\;{\left(b(x)\;o(x)\right)}^{\beta }\right]}^{\frac{1}{\beta }} \end{array}$$

(222) $$\begin{array}{cc} \; & =\log\min_{x}\left[b(x)\;o(x)\right], \end{array}$$

where (222) holds because, in the limit as β tends to −∞, the power mean tends to the minimum (since *p* is a PMF with p(x)>0 for all x∈X [15] (Chapter 8)).

We show (220) by contradiction. Assume that there exists a PMF *b* that does not satisfy (220), thus

(223) $$\begin{array}{c} b(x)\;o(x)>c \end{array}$$

for all x∈X. Then,

(224) $$\begin{array}{cc} 1 & =\sum_{x}b\left(x\right) \end{array}$$

(225) $$\begin{array}{cc} \; & >\sum_{x}\frac{c}{o(x)} \end{array}$$

(226) $$\begin{array}{cc} \; & =1, \end{array}$$

where (224) holds because *b* is a PMF; (225) follows from (223); and (226) follows from the definition of *c* in (204). Because 1>1 is impossible, such a *b* cannot exist, which establishes (220).

It is not difficult to see that (220) holds with equality if b(x)=c/o(x) for all x∈X. We therefore focus on establishing that if (220) holds with equality, then b(x)=c/o(x) for all x∈X. Observe first that, if (220) holds with equality, then, for all x∈X,

(227) $$\begin{array}{c} b(x)\;o(x)\geq c. \end{array}$$

We now claim that (227) holds with equality for all x∈X. Indeed, if this were not the case, then there would exist an $x^{\prime }\in X$ for which $b\left(x^{\prime }\right)\;o\left(x^{\prime }\right)>c$, thus (224)–(226) would hold, which would lead to a contradiction. Hence, if (220) holds with equality, then b(x)=c/o(x) for all x∈X. □

**Proposition** **9.** *Let* β≥1 *, and let b be a PMF. Then,*

(228) $$\begin{array}{c} U_{\beta }\leq \log\max_{x}\left[p{\left(x\right)}^{1/\beta }\;o\left(x\right)\right]. \end{array}$$

*Equality in* (228) *can be achieved by choosing* $b\left(x\right)=𝟙\left\{x=x^{\prime }\right\}$ *for some* $x^{\prime }\in X$ *satisfying*

(229) $$\begin{array}{c} p{\left(x^{\prime }\right)}^{1/\beta }\;o\left(x^{\prime }\right)=\max_{x}\left[p{\left(x\right)}^{1/\beta }\;o\left(x\right)\right]. \end{array}$$

**Remark** **7.** *Proposition 9 implies that if* β≥1 *, then it is optimal to bet on a single horse. Unless* |X|=1 *, this is not the case when* β<1 *: When* β<1 *, an optimal betting strategy requires placing a bet on every horse. This follows from Theorem 9 and our assumption that* p(x) *and* o(x) *are all positive.*

**Proof** **of** **Proposition** **9.** Inequality (228) holds because

(230) $$\begin{array}{cc} U_{\beta } & =\frac{1}{\beta }\log\sum_{x}p\left(x\right)\;b{\left(x\right)}^{\beta }\;o{\left(x\right)}^{\beta } \end{array}$$

(231) $$\begin{array}{cc} \; & \leq \frac{1}{\beta }\log\sum_{x}p\left(x\right)\;b\left(x\right)\;o{\left(x\right)}^{\beta } \end{array}$$

(232) $$\begin{array}{cc} \; & \leq \frac{1}{\beta }\log\sum_{x}b\left(x\right)\cdot \max_{x^{\prime }\in X}\left[p\left(x^{\prime }\right)\;o{\left(x^{\prime }\right)}^{\beta }\right] \end{array}$$

(233) $$\begin{array}{cc} \; & =\frac{1}{\beta }\log\max_{x^{\prime }\in X}\left[p\left(x^{\prime }\right)\;o{\left(x^{\prime }\right)}^{\beta }\right] \end{array}$$

(234) $$\begin{array}{cc} \; & =\log\max_{x^{\prime }\in X}\left[p{\left(x^{\prime }\right)}^{1/\beta }\;o\left(x^{\prime }\right)\right], \end{array}$$

where (231) holds because b(x)∈[0,1] and β≥1, and (233) holds because *b* is a PMF. It is not difficult to see that (228) holds with equality if $b\left(x\right)=𝟙\left\{x=x^{\prime }\right\}$ for some $x^{\prime }\in X$ satisfying (229). □

**Proposition** **10.** *Let b be a PMF. Then,*

(235) $$\begin{array}{cc} \lim_{\beta \to +\infty }U_{\beta } & =\log\max_{x}\left[b(x)\;o(x)\right] \end{array}$$

(236) $$\begin{array}{cc} \; & \leq \log\max_{x}o\left(x\right). \end{array}$$

*Equality in* (236) *can be achieved by choosing* $b\left(x\right)=𝟙\left\{x=x^{\prime }\right\}$ *for some* $x^{\prime }\in X$ *satisfying*

(237) $$\begin{array}{c} o\left(x^{\prime }\right)=\max_{x}o\left(x\right). \end{array}$$

**Proof.**  Equation (235) holds because

(238) $$\begin{array}{cc} \lim_{\beta \to +\infty }U_{\beta } & =\lim_{\beta \to +\infty }\log{\left[\sum_{x}p\left(x\right)\;{\left(b(x)\;o(x)\right)}^{\beta }\right]}^{\frac{1}{\beta }} \end{array}$$

(239) $$\begin{array}{cc} \; & =\log\max_{x}\left[b(x)\;o(x)\right], \end{array}$$

where (239) holds because in the limit as β tends to +∞, the power mean tends to the maximum (since *p* is a PMF with p(x)>0 for all x∈X [15] (Chapter 8)). Inequality (236) holds because b(x)≤1 for all x∈X. It is not difficult to see that (236) holds with equality if $b\left(x\right)=𝟙\left\{x=x^{\prime }\right\}$ for some $x^{\prime }\in X$ satisfying (237). □

## 9. Horse Betting with Side Information

In this section, we study the horse betting problem where the gambler observes some side information *Y* before placing her bets. This setting leads to the conditional Rényi divergence $D_{\alpha }^{l}\left(\cdot \right)$ discussed in Section 5 (see Theorem 10). In addition, it provides a new operational meaning to the dependence measure $J_{\alpha }\left(X;Y\right)$ (see Theorem 11).

We adapt our notation as follows: The joint PMF of *X* and *Y* is denoted $p_{XY}$. (Recall that *X* denotes the winning horse.) We drop the assumption that the winning probabilities p(x) are positive, but we assume that p(y)>0 for all y∈Y. We continue to assume that the gambler invests all her wealth, so a betting strategy is now a conditional PMF $b_{X|Y}$, and the wealth relative *S* is

(240) $$\begin{array}{c} S≜b(X|Y)\;o(X). \end{array}$$

As in Section 8, define the constant

(241) $$\begin{array}{c} c≜{\left[\sum_{x}\frac{1}{o(x)}\right]}^{-1} \end{array}$$

and the PMF

(242) $$\begin{array}{c} r_{X}\left(x\right)≜\frac{c}{o(x)}. \end{array}$$

The following decomposition of the utility function $U_{\beta }$ parallels that of Theorem 9:

**Theorem** **10.** *Let* β∈(−∞,1) *. Then,*

(243) $$\begin{array}{c} U_{\beta }=\log c+D_{\;\frac{1}{1-\beta }\;}^{l}(p_{X|Y}\|r_{X}\left|p_{Y}\right)-D_{1-\beta }\left(g_{X|Y}^{\left(\beta \right)}g_{Y}^{\left(\beta \right)}\|b_{X|Y}g_{Y}^{\left(\beta \right)}\right), \end{array}$$

*where the conditional PMF* $g_{X|Y}^{\left(\beta \right)}$ *and the PMF* $g_{Y}^{\left(\beta \right)}$ *are given by*

(244) $$\begin{array}{cc} g_{X|Y}^{\left(\beta \right)}\left(x|y\right) & ≜\frac{p{\left(x|y\right)}^{\frac{1}{1-\beta }}\;o{\left(x\right)}^{\frac{\beta }{1-\beta }}}{\sum_{x^{\prime }}p{\left(x^{\prime }|y\right)}^{\frac{1}{1-\beta }}\;o{\left(x^{\prime }\right)}^{\frac{\beta }{1-\beta }}}, \end{array}$$

(245) $$\begin{array}{cc} g_{Y}^{\left(\beta \right)}\left(y\right) & ≜\frac{p\left(y\right)\;{\left[\sum_{x^{\prime }}p{\left(x^{\prime }|y\right)}^{\frac{1}{1-\beta }}\;o{\left(x^{\prime }\right)}^{\frac{\beta }{1-\beta }}\right]}^{1-\beta }}{\sum_{y^{\prime }}p\left(y^{\prime }\right)\;{\left[\sum_{x^{\prime }}p{\left(x^{\prime }|y^{\prime }\right)}^{\frac{1}{1-\beta }}\;o{\left(x^{\prime }\right)}^{\frac{\beta }{1-\beta }}\right]}^{1-\beta }}. \end{array}$$

*Thus, choosing* $b_{X|Y}=g_{X|Y}^{\left(\beta \right)}$ *uniquely maximizes* $U_{\beta }$ *among all conditional PMFs* $b_{X|Y}$ *.*

**Proof.**  We first show that $U_{\beta }$ is uniquely maximized by $g_{X|Y}^{\left(\beta \right)}$. The only term on the RHS of (243) that depends on $b_{X|Y}$ is $-D_{1-\beta }\left(g_{X|Y}^{\left(\beta \right)}g_{Y}^{\left(\beta \right)}\|b_{X|Y}g_{Y}^{\left(\beta \right)}\right)$. Because 1−β>0, this term is maximized if and only if $b_{X|Y}g_{Y}^{\left(\beta \right)}=g_{X|Y}^{\left(\beta \right)}g_{Y}^{\left(\beta \right)}$ (Proposition 1 (a)). By our assumptions that p(y)>0 for all y∈Y and o(x)>0 for all x∈X, we have $g_{Y}^{\left(\beta \right)}\left(y\right)>0$ for all y∈Y. Consequently, $b_{X|Y}g_{Y}^{\left(\beta \right)}=g_{X|Y}^{\left(\beta \right)}g_{Y}^{\left(\beta \right)}$ if and only if $b_{X|Y}=g_{X|Y}^{\left(\beta \right)}$.

Consider now (243) for β=0. For β=0, (243) reduces to

(246) $$\begin{array}{c} E\left[\log S\right]=\log c+D\left(p_{X|Y}p_{Y}\|r_{X}p_{Y}\right)-D\left(p_{X|Y}p_{Y}\|b_{X|Y}p_{Y}\right), \end{array}$$

and some algebra reveals that (246) holds.

We conclude with establishing (243) for β∈(−∞,0)∪(0,1). For β∈(−∞,0)∪(0,1),

(247) $$\begin{array}{c} U_{\beta }=\frac{1}{\beta }\log\sum_{x,\;y}p\left(x,y\right)\;b{\left(x|y\right)}^{\beta }\;o{\left(x\right)}^{\beta }. \end{array}$$

For every x∈X and every y∈Y,

(248) $$\begin{array}{cc} p\left(x,y\right)\;b{\left(x|y\right)}^{\beta }\;o{\left(x\right)}^{\beta } & =\sum_{y^{\prime }\in Y}p\left(y^{\prime }\right){\left[\;\sum_{x^{\prime }\in X}p{\left(x^{\prime }|y^{\prime }\right)}^{\frac{1}{1-\beta }}\;o{\left(x^{\prime }\right)}^{\frac{\beta }{1-\beta }}\right]}^{1-\beta }\cdot g_{Y}^{\left(\beta \right)}\left(y\right)\;g_{X|Y}^{\left(\beta \right)}{\left(x|y\right)}^{1-\beta }\;b{\left(x|y\right)}^{\beta }, \end{array}$$

which follows from (244) and (245). Now, (243) holds because

$$\begin{array}{cc} U_{\beta } & =\frac{1}{\beta }\log\sum_{y^{\prime }\in Y}p\left(y^{\prime }\right){\left[\;\sum_{x^{\prime }\in X}p{\left(x^{\prime }|y^{\prime }\right)}^{\frac{1}{1-\beta }}\;o{\left(x^{\prime }\right)}^{\frac{\beta }{1-\beta }}\right]}^{1-\beta } \end{array}$$

(249) =+1βlog∑x,ygX|Y(β)(x|y)gY(β)(y)1−βb(x|y)gY(β)(y)β

(250) $$\begin{array}{cc} \; & =\frac{1}{\beta }\log\sum_{y^{\prime }\in Y}p\left(y^{\prime }\right){\left[\;\sum_{x^{\prime }\in X}p{\left(x^{\prime }|y^{\prime }\right)}^{\frac{1}{1-\beta }}\;o{\left(x^{\prime }\right)}^{\frac{\beta }{1-\beta }}\right]}^{1-\beta }-D_{1-\beta }\left(g_{X|Y}^{\left(\beta \right)}g_{Y}^{\left(\beta \right)}\|b_{X|Y}g_{Y}^{\left(\beta \right)}\right) \end{array}$$

(251) $$\begin{array}{cc} \; & =\log c+\frac{1}{\beta }\log\sum_{y^{\prime }\in Y}p\left(y^{\prime }\right){\left[\;\sum_{x^{\prime }\in X}p{\left(x^{\prime }|y^{\prime }\right)}^{\frac{1}{1-\beta }}\;r_{X}{\left(x^{\prime }\right)}^{\frac{-\beta }{1-\beta }}\right]}^{1-\beta }-D_{1-\beta }\left(g_{X|Y}^{\left(\beta \right)}g_{Y}^{\left(\beta \right)}\|b_{X|Y}g_{Y}^{\left(\beta \right)}\right) \end{array}$$

(252) $$\begin{array}{cc} \; & =\log c+D_{\;\frac{1}{1-\beta }\;}^{l}(p_{X|Y}\|r_{X}\left|p_{Y}\right)-D_{1-\beta }\left(g_{X|Y}^{\left(\beta \right)}g_{Y}^{\left(\beta \right)}\|b_{X|Y}g_{Y}^{\left(\beta \right)}\right), \end{array}$$

where (249) follows from (247) and (248) and the fact that $g_{Y}^{\left(\beta \right)}\left(y\right)=g_{Y}^{\left(\beta \right)}{\left(y\right)}^{1-\beta }\;g_{Y}^{\left(\beta \right)}{\left(y\right)}^{\beta }$; (250) follows by identifying the Rényi divergence; (251) follows from (242); and (252) follows by identifying the conditional Rényi divergence using (78). □

**Remark** **8.** *It follows from Theorem 10 that, if the gambler gambles optimally, then, for* β∈(−∞,1) *,*

(253) $$\begin{array}{c} U_{\beta }=\log c+D_{\;\frac{1}{1-\beta }\;}^{l}(p_{X|Y}\|r_{X}\left|p_{Y}\right). \end{array}$$

*Operationally, it is clear that preprocessing the side information cannot increase the gambler’s utility, i.e., that, for every conditional PMF* $p_{Y^{\prime }|Y}$ *,*

(254) $$\begin{array}{c} D_{\;\frac{1}{1-\beta }\;}^{l}(p_{X|Y^{\prime }}\|r_{X}|p_{Y^{\prime }})\leq D_{\;\frac{1}{1-\beta }\;}^{l}(p_{X|Y}\|r_{X}\left|p_{Y}\right), \end{array}$$

*where* $p_{X|Y^{\prime }}$ *and* $p_{Y^{\prime }}$ *are derived from the joint PMF* $p_{XYY^{\prime }}$ *given by*

(255) $$\begin{array}{c} p_{XYY^{\prime }}\left(x,y,y^{\prime }\right)=p_{Y}\left(y\right)\;p_{X|Y}\left(x|y\right)\;p_{Y^{\prime }|Y}\left(y^{\prime }|y\right). \end{array}$$

*This provides the intuition for Theorem 6, where* (254) *is shown directly.*

*The extreme case is when the preprocessing maps the side information to a constant and hence leads to the case where the side information is absent. In this case,* $Y^{\prime }$ *is deterministic and* $p_{X|Y^{\prime }}$ *equals* $p_{X}$ *. Theorem 9 and Theorem 10 then lead to the following relation between the conditional and unconditional Rényi divergence:*

(256) $$\begin{array}{c} D_{\;\frac{1}{1-\beta }\;}\left(p_{X}\|r_{X}\right)\leq D_{\;\frac{1}{1-\beta }\;}^{l}(p_{X|Y}\|r_{X}\left|p_{Y}\right), \end{array}$$

*where the marginal PMF* $p_{X}$ *is given by*

(257) $$\begin{array}{c} p_{X}\left(x\right)=\sum_{y}p_{XY}\left(x,y\right). \end{array}$$

*This motivates Corollary 3, where (256) is derived from (254).*

The last result of this section provides a new operational meaning to the Lapidoth–Pfister mutual information $J_{\alpha }\left(X;Y\right)$: assuming that β∈(−∞,1) and that the gambler knows the winning probabilities, $J_{1/(1-\beta )}\left(X;Y\right)$ measures how much the side information that is available to the gambler but not the bookmaker increases the gambler’s smallest guaranteed utility for a fixed level of fairness *c*. To see this, consider first the setting without side information. By Theorem 9, the gambler chooses $b=g^{\left(\beta \right)}$ to maximize her utility, where $g^{\left(\beta \right)}$ is defined in (207). Then, using the nonnegativity of the Rényi divergence (Proposition 1 (a)), the following lower bound on the gambler’s utility follows from (206):

(258) $$\begin{array}{c} U_{\beta }\geq \log c. \end{array}$$

We call the RHS of (258) the smallest guaranteed utility for a fixed level of fairness *c* because (258) holds with equality if the bookmaker chooses the odds inversely proportional to the winning probabilities. Comparing (258) with (259) below, we see that the difference due to the side information is $J_{1/(1-\beta )}\left(X;Y\right)$. Note that $J_{1/(1-\beta )}\left(X;Y\right)$ is typically not the difference between the utility with and without side information; this is because the odds for which (258) and (259) hold with equality are typically not the same.

**Theorem** **11.** *Let* β∈(−∞,1) *. If* $b_{X|Y}$ *is equal to* $g_{X|Y}^{\left(\beta \right)}$ *from Theorem 10, then*

(259) $$\begin{array}{c} U_{\beta }\geq \log c+J_{\;\frac{1}{1-\beta }\;}\left(X;Y\right). \end{array}$$

*Moreover, for every*c>0*, there exist odds*o:X→(0,∞)*such that* (259) *holds with equality.*

**Proof.**  For this choice of $b_{X|Y}$, (259) holds because

(260) $$\begin{array}{cc} U_{\beta } & =\log c+D_{\;\frac{1}{1-\beta }\;}^{l}(p_{X|Y}\|r_{X}\left|p_{Y}\right) \end{array}$$

(261) $$\begin{array}{cc} \; & \geq \log c+\min_{{\tilde{r}}_{X}\in P\left(X\right)}D_{\;\frac{1}{1-\beta }\;}^{l}(p_{X|Y}\|{\tilde{r}}_{X}\left|p_{Y}\right) \end{array}$$

(262) $$\begin{array}{cc} \; & =\log c+J_{\;\frac{1}{1-\beta }\;}\left(X;Y\right), \end{array}$$

where (260) follows from Theorem 10, and (262) follows from Proposition 5.

Fix now c>0, let ${\tilde{r}}_{X}^{*}$ achieve the minimum on the RHS of (261), and choose the odds

(263) $$\begin{array}{c} o\left(x\right)=\frac{c}{{\tilde{r}}_{X}^{*}\left(x\right)}. \end{array}$$

Then, (261) holds with equality because $r_{X}={\tilde{r}}_{X}^{*}$ by (241) and (242). □

## 10. Horse Betting with Part of the Money

In this section, we treat the possibility that the gambler does not invest all her wealth. We restrict ourselves to the setting without side information and to β∈(−∞,0)∪(0,1). (For the case β=0, see [47] (Section 10.5).) We assume that p(x)>0 and o(x)>0 for all x∈X. Denote by b(0) the fraction of her wealth that the gambler does not use for betting. (We assume 0∉X.) Then, b:X∪{0}→[0,1] is a PMF, and the wealth relative *S* is the random variable

(264) $$\begin{array}{c} S≜b(0)+b(X)\;o(X). \end{array}$$

As in Section 8, define the constant

(265) $$\begin{array}{c} c≜{\left[\sum_{x}\frac{1}{o(x)}\right]}^{-1}. \end{array}$$

We treat the cases c<1 and c≥1 separately, starting with the latter. If c≥1, then it is optimal to invest all the money:

**Proposition** **11.** *Assume*c≥1*, let*β∈R*, and let b be a PMF on*X∪{0}*with utility*$U_{\beta }$*. Then, there exists a PMF*$b^{\prime }$ on X∪{0} with $b^{\prime }\left(0\right)=0$ *and utility* $U_{\beta }^{\prime }\geq U_{\beta }$*.*

**Proof.**  Choose the PMF $b^{\prime }$ as follows:

(266) $$\begin{array}{c} b^{\prime }\left(x\right)=\left\{\begin{array}{cc} \frac{c}{o(x)}\cdot b\left(0\right)+b\left(x\right) & \mathrm{if}\;x\in X,\\ 0 & \mathrm{if}\;x=0. \end{array}\right. \end{array}$$

Then, for every x∈X,

(267) $$\begin{array}{cc} b^{\prime }\left(0\right)+b^{\prime }\left(x\right)\;o\left(x\right) & =c\cdot b(0)+b(x)\;o(x) \end{array}$$

(268) $$\begin{array}{cc} \; & \geq b(0)+b(x)\;o(x), \end{array}$$

where (268) holds because c≥1 by assumption. For β>0, $U_{\beta }^{\prime }\geq U_{\beta }$ holds because (268) implies $E\left[S^{{}^{\prime }\beta }\right]\geq E\left[S^{\beta }\right]$. For β<0 and β=0, $U_{\beta }^{\prime }\geq U_{\beta }$ follows similarly from (268). □

On the other hand, if β<1 and the odds are subfair, i.e., if c<1, then Claim (c) of the following theorem shows that investing all the money is not optimal:

**Theorem** **12.** *Assume* c<1 *, let* β∈(−∞,0)∪(0,1) *, and let* $b^{*}$ *be a PMF on* X∪{0} *that maximizes* $U_{\beta }$ *among all PMFs b. Defining*

(269) $$\begin{array}{cc} S & ≜\{x\in X:b^{*}\left(x\right)>0\}, \end{array}$$

(270) $$\begin{array}{cc} \Gamma & ≜\frac{1-\sum_{x\in S}p\left(x\right)}{1-\sum_{x\in S}\frac{1}{o(x)}}, \end{array}$$

(271) $$\begin{array}{cc} \gamma (x) & ≜\max\;\left\{0,\;\Gamma^{\frac{1}{\beta -1}}\;p{\left(x\right)}^{\frac{1}{1-\beta }}\;o{\left(x\right)}^{\frac{\beta }{1-\beta }}-\frac{1}{o(x)}\right\}\;\forall \;x\in X, \end{array}$$

*the following claims hold:*

*(a)*  *Both the numerator and denominator on the RHS of* (270) *are positive, so* Γ *is well-defined and positive.*
*(b)*  *For every* x∈X *,*
  (272) $$\begin{array}{c} b^{*}\left(x\right)=\gamma \left(x\right)\;b^{*}\left(0\right). \end{array}$$
*(c)*  *The quantity* $b^{*}\left(0\right)$ *satisfies*
  (273) $$\begin{array}{c} b^{*}\left(0\right)=\frac{1}{1+\sum_{x\in X}\gamma \left(x\right)}. \end{array}$$
*In particular,* $b^{*}\left(0\right)>0$ *.*

Claim (b) implies that for every x∈X, $b^{*}\left(x\right)>0$ if and only if p(x)o(x)>Γ. Ordering the elements $x_{1},x_{2},\ldots$ of X such that $p\left(x_{1}\right)\;o\left(x_{1}\right)\geq p\left(x_{2}\right)\;o\left(x_{2}\right)\geq \ldots$, the set S thus has a special structure: it is either empty or equal to $\left\{x_{1},x_{2},\ldots ,x_{k}\right\}$ for some integer *k*. To maximize $U_{\beta }$, the following procedure can be used: for every S with the above structure, compute the corresponding *b* according to (270)–(273); and from these *b*’s, take one that maximizes $U_{\beta }$. This procedure leads to an optimal solution: an optimal solution $b^{*}$ exists because we are optimizing a continuous function over a compact set, and $b^{*}$ corresponds to a set S that will be considered by the procedure.

**Proof** **of** **Theorem** **12.** The proof is based on the Karush–Kuhn–Tucker conditions. By separately considering the cases β∈(0,1) and β<0, we first show that, for β∈(−∞,0)∪(0,1), a strategy b(·) is optimal if and only if the following conditions are satisfied for some μ∈R:

(274) $$\begin{array}{c} \sum_{x\in X}p\left(x\right)\;{\left(b(0)+b(x)\;o(x)\right)}^{\beta -1}\left\{\begin{array}{cc} =\mu & \mathrm{if}\;b(0)>0,\\ \leq \mu & \mathrm{if}\;b(0)=0, \end{array}\right. \end{array}$$

and, for every x∈X,

(275) $$\begin{array}{c} p\left(x\right)\;o\left(x\right)\;{\left(b(0)+b(x)\;o(x)\right)}^{\beta -1}\left\{\begin{array}{cc} =\mu & \mathrm{if}\;b(x)>0,\\ \leq \mu & \mathrm{if}\;b(x)=0. \end{array}\right. \end{array}$$

Consider first β∈(0,1), and define the function τ:P(X∪{0})→R,

(276) $$\begin{array}{c} \tau \left(b\right)≜\sum_{x\in X}p\left(x\right)\;{\left(b(0)+b(x)\;o(x)\right)}^{\beta }. \end{array}$$

Since β>0 and since the logarithm is an increasing function, maximizing $U_{\beta }=\frac{1}{\beta }\log E\left[S^{\beta }\right]$ over *b* is equivalent to maximizing τ(b). Observe that τ is concave, thus, by the Karush–Kuhn–Tucker conditions [11] (Theorem 4.4.1), it is maximized by a PMF *b* if and only if there exists a λ∈R such that (i) for all x∈X∪{0} with b(x)>0,

(277) $$\begin{array}{c} \frac{\partial \tau }{\partial b(x)}\left(b\right)=\lambda , \end{array}$$

and (ii) for all x∈X∪{0} with b(x)=0,

(278) $$\begin{array}{c} \frac{\partial \tau }{\partial b(x)}\left(b\right)\leq \lambda . \end{array}$$

Henceforth, we use the following notation: to designate that (i) and (ii) both hold, we write

(279) $$\begin{array}{c} \frac{\partial \tau }{\partial b(x)}\left(b\right)\left\{\begin{array}{cc} =\lambda & \mathrm{if}\;b(x)>0,\\ \leq \lambda & \mathrm{if}\;b(x)=0. \end{array}\right. \end{array}$$

Dividing both sides of (279) by β>0 and defining $\mu ≜\frac{\lambda }{\beta }$, we obtain that (279) is equivalent to

(280) $$\begin{array}{c} \frac{1}{\beta }\cdot \frac{\partial \tau }{\partial b(x)}\left(b\right)\left\{\begin{array}{cc} =\mu & \mathrm{if}\;b(x)>0,\\ \leq \mu & \mathrm{if}\;b(x)=0. \end{array}\right. \end{array}$$

Now, (280) translates to (274) for x=0 and to (275) for x∈X.

Consider now β<0, and define τ as in (276). Then, because β<0, maximizing $U_{\beta }=\frac{1}{\beta }\log E\left[S^{\beta }\right]$ is equivalent to minimizing τ. The function τ is convex, thus Inequality (278) is reversed. Dividing by β<0 again reverses the inequalities, thus (280), (274), and (275) continue to hold for β<0.

Having established that, for all β∈(−∞,0)∪(0,1), a strategy *b* is optimal if and only if (274) and (275) hold, we next continue with the proof. Let β∈(−∞,0)∪(0,1), and let $b^{*}$ be a PMF on X∪{0} that maximizes $U_{\beta }$. By the above discussion, (274) and (275) are satisfied by $b^{*}$ for some μ∈R. The LHS of (274) is positive, so μ>0. We now show that for all x∈X,

(281) $$\begin{array}{c} b^{*}\left(x\right)=\max\;\left\{0,\;{\left[\frac{p\left(x\right)\;o{\left(x\right)}^{\beta }}{\mu }\right]}^{\frac{1}{1-\beta }}-\frac{b^{*}\left(0\right)}{o(x)}\right\}. \end{array}$$

To this end, fix x∈X. If $b^{*}\left(x\right)>0$, then (275) implies

(282) $$\begin{array}{c} b^{*}\left(x\right)={\left[\frac{p\left(x\right)\;o{\left(x\right)}^{\beta }}{\mu }\right]}^{\frac{1}{1-\beta }}-\frac{b^{*}\left(0\right)}{o(x)}, \end{array}$$

and the RHS of (282) is equal to the RHS of (281) because, being equal to $b^{*}\left(x\right)$, it is positive. If $b^{*}\left(x\right)=0$, then (275) implies

(283) $$\begin{array}{c} {\left[\frac{p\left(x\right)\;o{\left(x\right)}^{\beta }}{\mu }\right]}^{\frac{1}{1-\beta }}-\frac{b^{*}\left(0\right)}{o(x)}\leq 0, \end{array}$$

so the RHS of (281) is zero and (281) hence holds.

Having established (281), we next show that $b^{*}\left(\hat{x}\right)=0$ for some $\hat{x}\in X$. For a contradiction, assume that $b^{*}\left(x\right)>0$ for all x∈X. Then,

(284) $$\begin{array}{cc} \sum_{x\in X}p\left(x\right)\;{\left(b^{*}\left(0\right)+b^{*}\left(x\right)\;o\left(x\right)\right)}^{\beta -1} & =\mu \sum_{x\in X}\frac{1}{o(x)} \end{array}$$

(285) $$\begin{array}{cc} \; & >\mu , \end{array}$$

where (284) follows from (275), and (285) holds because c<1 by assumption. However, this is impossible: (285) contradicts (274).

Let now $\hat{x}\in X$ be such that $b^{*}\left(\hat{x}\right)=0$. Then, by (281),

(286) $$\begin{array}{c} {\left[\frac{p\left(\hat{x}\right)\;o{\left(\hat{x}\right)}^{\beta }}{\mu }\right]}^{\frac{1}{1-\beta }}-\frac{b^{*}\left(0\right)}{o(\hat{x})}\leq 0. \end{array}$$

Because $p(\hat{x})$ and $o(\hat{x})$ are positive, this implies $b^{*}\left(0\right)>0$. Thus, by (274),

(287) $$\begin{array}{c} \sum_{x\in X}p\left(x\right)\;{\left(b^{*}\left(0\right)+b^{*}\left(x\right)\;o\left(x\right)\right)}^{\beta -1}=\mu . \end{array}$$

Splitting the sum on the LHS of (287) depending on whether $b^{*}\left(x\right)>0$ or $b^{*}\left(x\right)=0$, we obtain

(288) $$\begin{array}{cc} \mu & =\sum_{x\in S}p\left(x\right)\;{\left(b^{*}\left(0\right)+b^{*}\left(x\right)\;o\left(x\right)\right)}^{\beta -1}+\sum_{x\notin S}p\left(x\right)\;{\left(b^{*}\left(0\right)+b^{*}\left(x\right)\;o\left(x\right)\right)}^{\beta -1} \end{array}$$

(289) $$\begin{array}{cc} \; & =\sum_{x\in S}\frac{\mu }{o(x)}+\sum_{x\notin S}p\left(x\right)\;b^{*}{\left(0\right)}^{\beta -1} \end{array}$$

(290) $$\begin{array}{cc} \; & =\mu \sum_{x\in S}\frac{1}{o(x)}+b^{*}{\left(0\right)}^{\beta -1}\;\left[1-\sum_{x\in S}p\left(x\right)\right], \end{array}$$

where (289) follows from (275). Rearranging (290), we obtain

(291) $$\begin{array}{c} \mu \left[1-\sum_{x\in S}\frac{1}{o(x)}\right]=b^{*}{\left(0\right)}^{\beta -1}\;\left[1-\sum_{x\in S}p\left(x\right)\right]. \end{array}$$

Recall that μ>0 and $b^{*}\left(0\right)>0$. In addition, $1-\sum_{x\in S}p\left(x\right)>0$ because $b^{*}\left(\hat{x}\right)=0$ and hence $\hat{x}\notin S$. Thus, $1-\sum_{x\in S}\frac{1}{o(x)}>0$, so both the numerator and denominator in the definition of Γ in (270) are positive, which establishes Claim (a), namely that Γ is well-defined and positive.

To establish Claim (b), note that (291) and (270) imply that μ is given by

(292) $$\begin{array}{c} \mu =b^{*}{\left(0\right)}^{\beta -1}\;\Gamma , \end{array}$$

which, when substituted into (281), yields (272).

We conclude by proving Claim (c). Because $b^{*}$ is a PMF on X∪{0},

(293) $$\begin{array}{cc} 1 & =b^{*}\left(0\right)+\sum_{x\in X}b^{*}\left(x\right) \end{array}$$

(294) $$\begin{array}{cc} \; & =b^{*}\left(0\right)\;\left[1+\sum_{x\in X}\gamma \left(x\right)\right], \end{array}$$

where (294) follows from (272). Rearranging (294) yields (273). □

## 11. Universal Betting for IID Races

In this section, we present a universal gambling strategy for IID races that requires neither knowledge of the winning probabilities nor of the parameter β of the utility function and yet asymptotically maximizes the utility function for all PMFs *p* and all β∈R. Consider *n* consecutive horse races, where the winning horse in the *i*th race is denoted $X_{i}$ for i∈{1,…,n}. We assume that $X_{1},\ldots ,X_{n}$ are IID according to the PMF *p*, where p(x)>0 for all x∈X. In every race, the bookmaker offers the same odds o:X→(0,∞), and the gambler spends all her wealth placing bets on the horses. The gambler plays race-after-race, i.e., before placing bets for a race, she is revealed the winning horse of the previous race and receives the money from the bookmaker. Her betting strategy is hence a sequence of conditional PMFs $\left(b_{X_{1}},b_{X_{2}|X_{1}},b_{X_{3}|X_{1}X_{2}},\ldots ,b_{X_{n}|X_{1}X_{2}\cdots X_{n-1}}\right)$. The wealth relative is the random variable

(295) $$\begin{array}{c} S_{n}≜\prod_{i=1}^{n}b\left(X_{i}|X_{1},\ldots ,X_{i-1}\right)\;o\left(X_{i}\right). \end{array}$$

We seek betting strategies that maximize the utility function

(296) $$\begin{array}{c} U_{\beta ,n}≜\left\{\begin{array}{cc} \frac{1}{\beta }\log E\left[S_{n}^{\beta }\right] & \mathrm{if}\;\beta \neq 0,\\ E[\log S_{n}] & \mathrm{if}\;\beta =0. \end{array}\right. \end{array}$$

We first establish that to maximize $U_{\beta ,n}$ for a fixed β∈R, it suffices to use the same betting strategy in every race; see Theorem 13. We then show that the individual-sequence-universal strategy by Cover–Ordentlich [48] allows to asymptotically achieve the same normalized utility without knowing *p* or β (see Theorem 14).

For a fixed β∈R, let the PMF $b^{*}$ be a betting strategy that maximizes the single-race utility $U_{\beta }$ discussed in Section 8, and denote by $U_{\beta }^{*}$ the utility associated with $b^{*}$. Using the same betting strategy $b^{*}$ over *n* races leads to the utility $U_{\beta ,n}$, and it follows from (295) and (296) that

(297) $$\begin{array}{c} U_{\beta ,n}=n\;U_{\beta }^{*}. \end{array}$$

As we show next, $n\;U_{\beta }^{*}$ is the maximum utility that can be achieved among all betting strategies:

**Theorem** **13.** *Let* β∈R *, and let* $\left(b_{X_{1}},b_{X_{2}|X_{1}},b_{X_{3}|X_{1}X_{2}},\ldots ,b_{X_{n}|X_{1}X_{2}\cdots X_{n-1}}\right)$ *be a sequence of conditional PMFs. Then,*

(298) $$\begin{array}{c} U_{\beta ,n}\leq n\;U_{\beta }^{*}. \end{array}$$

**Proof.**  We show (298) for β>0; analogous arguments establish (298) for β<0 and β=0. We prove (298) by induction on *n*. For n=1, (298) holds because $U_{\beta }^{*}$ is the maximum single-race utility. Assume now n≥2 and that (298) is valid for n−1. For β>0, (298) holds because

(299) $$\begin{array}{cc} U_{\beta ,n} & =\frac{1}{\beta }\log E\left[S_{n}^{\beta }\right] \end{array}$$

(300) $$\begin{array}{cc} \; & =\frac{1}{\beta }\log\sum_{x_{1},\ldots ,x_{n}}P\left(x_{1}\right)\cdots P\left(x_{n}\right)\prod_{i=1}^{n}b{\left(x_{i}|x^{i-1}\right)}^{\beta }\;o{\left(x_{i}\right)}^{\beta } \end{array}$$

(301) $$\begin{array}{cc} \; & =\frac{1}{\beta }\log\sum_{x_{1},\ldots ,x_{n-1}}P\left(x_{1}\right)\cdots P\left(x_{n-1}\right)\;\left[\prod_{i=1}^{n-1}b{\left(x_{i}|x^{i-1}\right)}^{\beta }\;o{\left(x_{i}\right)}^{\beta }\right]\sum_{x_{n}}P\left(x_{n}\right)\;b{\left(x_{n}|x^{n-1}\right)}^{\beta }\;o{\left(x_{n}\right)}^{\beta } \end{array}$$

(302) $$\begin{array}{cc} \; & \leq \frac{1}{\beta }\log\sum_{x_{1},\ldots ,x_{n-1}}P\left(x_{1}\right)\cdots P\left(x_{n-1}\right)\;\left[\prod_{i=1}^{n-1}b{\left(x_{i}|x^{i-1}\right)}^{\beta }\;o{\left(x_{i}\right)}^{\beta }\right]\max_{b\in P(X)}\sum_{x_{n}}P\left(x_{n}\right)\;b{\left(x_{n}\right)}^{\beta }\;o{\left(x_{n}\right)}^{\beta } \end{array}$$

(303) $$\begin{array}{cc} \; & =\frac{1}{\beta }\log\sum_{x_{1},\ldots ,x_{n-1}}P\left(x_{1}\right)\cdots P\left(x_{n-1}\right)\;\left[\prod_{i=1}^{n-1}b{\left(x_{i}|x^{i-1}\right)}^{\beta }\;o{\left(x_{i}\right)}^{\beta }\right]\sum_{x_{n}}P\left(x_{n}\right)\;b^{*}{\left(x_{n}\right)}^{\beta }\;o{\left(x_{n}\right)}^{\beta } \end{array}$$

(304) $$\begin{array}{cc} \; & =U_{\beta ,n-1}+U_{\beta }^{*} \end{array}$$

(305) $$\begin{array}{cc} \; & \leq \left(n-1\right)\;U_{\beta }^{*}+U_{\beta }^{*} \end{array}$$

(306) $$\begin{array}{cc} \; & =n\;U_{\beta }^{*}, \end{array}$$

where (303) holds because $b^{*}$ maximizes the single-race utility $U_{\beta }$, and (305) holds because (298) is valid for n−1. □

In portfolio theory, Cover–Ordentlich [48] (Definition 1) proposed a universal strategy. Adapted to our setting, it leads to the following sequence of conditional PMFs:

(307) $$\begin{array}{c} \hat{b}\left(x_{i}|x^{i-1}\right)=\frac{\int_{b\in P(X)}b\left(x_{i}\right)\;S_{i-1}\left(b,x^{i-1}\right)\;d\;\mu \left(b\right)}{\int_{b\in P(X)}S_{i-1}\left(b,x^{i-1}\right)\;d\;\mu \left(b\right)}, \end{array}$$

where i∈{1,2,…}; μ is the Dirichlet(1/2,…,1/2) distribution on P(X); $S_{0}\left(b,x^{0}\right)≜1$; and

(308) $$\begin{array}{c} S_{i}\left(b,x^{i}\right)≜\prod_{j=1}^{i}b\left(x_{j}\right)\;o\left(x_{j}\right). \end{array}$$

This strategy depends neither on the winning probabilities *p* nor on the parameter β. Denoting the utility (296) associated with the strategy $\hat{b}\left(x_{i}|x^{i-1}\right)$ by ${\hat{U}}_{\beta ,n}$, we have the following result:

**Theorem** **14.** *For every* β∈R *,*

(309) $$\begin{array}{cc} n\;U_{\beta }^{*}-\log2-\frac{|X|-1}{2}\log\left(n+1\right) & \leq {\hat{U}}_{\beta ,n} \end{array}$$

(310) $$\begin{array}{cc} \; & \leq n\;U_{\beta }^{*}. \end{array}$$

*Hence,*

(311) $$\begin{array}{c} \lim_{n\to \infty }\frac{1}{n}\;{\hat{U}}_{\beta ,n}=U_{\beta }^{*}. \end{array}$$

**Proof.**  Inequality (310) follows from Theorem 13; and (311) follows from (309) and (310) and the sandwich theorem. It thus remains to establish (309): We do so for β>0; analogous arguments establish (309) for β<0 and β=0. For a fixed sequence $x^{n}\in X^{n}$, let $\tilde{b}$ be a PMF on X that maximizes $S_{n}\left(b,x^{n}\right)$, and denote the wealth relative in (295) associated with using $\tilde{b}$ in every race by ${\tilde{S}}_{n}\left(x^{n}\right)$, thus

(312) $$\begin{array}{c} {\tilde{S}}_{n}\left(x^{n}\right)=\max_{b\in P(X)}\prod_{i=1}^{n}b\left(x_{i}\right)\;o\left(x_{i}\right). \end{array}$$

Let ${\hat{S}}_{n}\left(x^{n}\right)$ denote the wealth relative in (295) associated with the strategy $\hat{b}\left(x_{i}|x^{i-1}\right)$ and the sequence $x^{n}$. Using [48] (Theorem 2) it follows that, for every $x^{n}\in X^{n}$,

(313) $$\begin{array}{c} {\hat{S}}_{n}\left(x^{n}\right)\geq \frac{1}{2\;{\left(n+1\right)}^{(|X|-1)/2}}\;{\tilde{S}}_{n}\left(x^{n}\right). \end{array}$$

This implies that (309) holds for β>0 because

(314) $$\begin{array}{cc} {\hat{U}}_{\beta ,n} & =\frac{1}{\beta }\log E\left[{\hat{S}}_{n}{\left(X^{n}\right)}^{\beta }\right] \end{array}$$

(315) $$\begin{array}{cc} \; & \geq \frac{1}{\beta }\log E\left[{\tilde{S}}_{n}{\left(X^{n}\right)}^{\beta }\right]-\log2-\frac{|X|-1}{2}\log\left(n+1\right) \end{array}$$

(316) $$\begin{array}{cc} \; & \geq \frac{1}{\beta }\log\sum_{x_{1},\ldots ,x_{n}}P\left(x_{1}\right)\cdots P\left(x_{n}\right)\prod_{i=1}^{n}b^{*}{\left(x_{i}\right)}^{\beta }\;o{\left(x_{i}\right)}^{\beta }-\log2-\frac{|X|-1}{2}\log\left(n+1\right) \end{array}$$

(317) $$\begin{array}{cc} \; & =n\;U_{\beta }^{*}-\log2-\frac{|X|-1}{2}\log\left(n+1\right), \end{array}$$

where (315) follows from (313), and (316) follows from (312). □

**Remark** **9.** *As discussed in Section 8, the optimal single-race betting strategy varies significantly with different values of β, thus it might be a bit surprising that the Cover–Ordentlich strategy is not only universal with respect to the winning probabilities, but also with respect to β. This is due to the following two reasons: First, for fixed winning probabilities and a fixed β, it is optimal to use the same betting strategy in every race (see Theorem 13). Second, for every*$x^{n}\in X^{n}$*, the wealth relative of the Cover–Ordentlich strategy is not much worse than that of using the same strategy*b(·)*in every race, irrespective of*b(·)*(see* (313)*). Hence, irrespective of the optimal single-race betting strategy, the Cover–Ordentlich strategy is able to asymptotically achieve the same normalized utility.*

## Appendix A. Proof of Proposition 1

These properties mostly follow from van Erven–Harremoës [8]:

(a) See [8] (Theorem 8).
(b) This follows from the definitions in (4) and (21)–(23) and the conventions in (20).
(c) This follows from [8] (Theorem 7) and the fact that $\lim_{\alpha \to 1}D_{\alpha }\left(P\|Q\right)=D\left(P\|Q\right)$ by L’Hôpital’s rule. (Note that $\alpha \mapsto D_{\alpha }\left(P\|Q\right)$ does not need to be continuous at α=1 when the alphabets are not finite; see the discussion after [8] (Equation (18)).)
(d) See [8] (Theorem 3).
(e) Let $\alpha ,\alpha^{\prime }\in \left(0,\infty \right)$ satisfy $\alpha \leq \alpha^{\prime }$. Then,
  (A1) $$\begin{array}{cc} \frac{1-\alpha }{\alpha }\;D_{\alpha }\left(P\|Q\right) & =D_{1-\alpha }\left(Q\|P\right) \end{array}$$
  (A2) $$\begin{array}{cc} \; & \geq D_{1-\alpha^{\prime }}\left(Q\|P\right) \end{array}$$
  (A3) $$\begin{array}{cc} \; & =\frac{1-\alpha^{\prime }}{\alpha^{\prime }}\;D_{\alpha^{\prime }}\left(P\|Q\right), \end{array}$$
  where (A1) and (A3) follow from [8] (Lemma 10), and (A2) holds because the Rényi divergence, extended to negative orders, is nondecreasing ([8] (Theorem 39)).
(f) See [8] (Corollary 2).
(g) For α∈(0,∞),
  (A4) $$\begin{array}{cc} \left(\alpha -1\right)\;D_{1/\alpha }\left(P\|Q\right) & =\alpha \;\left(1-\frac{1}{\alpha }\right)\;D_{1/\alpha }\left(P\|Q\right) \end{array}$$
  (A5) $$\begin{array}{cc} \; & =\alpha \underset{R}{\inf}\;\left[\frac{1}{\alpha }\;D\left(R\|P\right)+\left(1-\frac{1}{\alpha }\right)\;D\left(R\|Q\right)\right] \end{array}$$
  (A6) $$\begin{array}{cc} \; & =\underset{R}{\inf}\;\left[D(R\|P)+(\alpha -1)\;D(R\|Q)\right], \end{array}$$
  where (A5) follows from [8] (Theorem 30). Hence, $\left(\alpha -1\right)\;D_{1/\alpha }\left(P\|Q\right)$ is concave in α because the expression in square brackets on the RHS of (A6) is concave in α for every *R* and because the pointwise infimum preserves the concavity.
(h) See [8] (Theorem 9).

## Appendix B. Proof of Theorem 1

Beginning with (29),

(A7) $$\begin{array}{cc} D_{\alpha }^{c}(P_{Y^{\prime }|X}\|Q_{Y^{\prime }|X}\left|P_{X}\right) & =\sum_{x\in \mathrm{supp}(P_{X})}P\left(x\right)\;D_{\alpha }\left(P_{Y^{\prime }|X=x}\|Q_{Y^{\prime }|X=x}\right) \end{array}$$

(A8) $$\begin{array}{cc} \; & \leq \sum_{x\in \mathrm{supp}(P_{X})}P\left(x\right)\;D_{\alpha }\left(P_{Y|X=x}\|Q_{Y|X=x}\right) \end{array}$$

(A9) $$\begin{array}{cc} \; & =D_{\alpha }^{c}(P_{Y|X}\|Q_{Y|X}\left|P_{X}\right), \end{array}$$

where (A8) follows by applying, separately for every $x\in \mathrm{supp}(P_{X})$, Proposition 1 (h) with the conditional PMF $A_{Y^{\prime }|Y,X=x}$.

## Appendix C. Proof of Theorem 2

We show (43) for α∈(0,1); the claim then extends to α∈[0,1] by the continuity of $D_{\alpha }^{c}\left(\cdot \right)$ in α (Proposition 2 (c)). Let α∈(0,1). Keeping in mind that α−1<0, (43) holds because

$$\begin{array}{cc} \; & \left(\alpha -1\right)\;D_{\alpha }^{c}(P_{Y|X^{\prime }}\|Q_{Y|X^{\prime }}\left|P_{X^{\prime }}\right) \end{array}$$

(A10) $$\begin{array}{cc} \; & \;=\sum_{x^{\prime }\in \mathrm{supp}\left(P_{X^{\prime }}\right)}P_{X^{\prime }}\left(x^{\prime }\right)\log\sum_{y}P_{Y|X^{\prime }}{\left(y|x^{\prime }\right)}^{\alpha }\;Q_{Y|X^{\prime }}{\left(y|x^{\prime }\right)}^{1-\alpha } \end{array}$$

(A11) $$\begin{array}{cc} \; & \;=\sum_{x^{\prime }\in \mathrm{supp}\left(P_{X^{\prime }}\right)}P_{X^{\prime }}\left(x^{\prime }\right)\log\sum_{y}{\left[\sum_{x}B_{X|X^{\prime }}\left(x|x^{\prime }\right)\;P_{Y|X}\left(y|x\right)\right]}^{\alpha }\;{\left[\sum_{x}B_{X|X^{\prime }}\left(x|x^{\prime }\right)\;Q_{Y|X}\left(y|x\right)\right]}^{1-\alpha } \end{array}$$

(A12) $$\begin{array}{cc} \; & \;\geq \sum_{x^{\prime }\in \mathrm{supp}\left(P_{X^{\prime }}\right)}P_{X^{\prime }}\left(x^{\prime }\right)\log\sum_{y}\sum_{x}B_{X|X^{\prime }}\left(x|x^{\prime }\right)\;P_{Y|X}{\left(y|x\right)}^{\alpha }\;Q_{Y|X}{\left(y|x\right)}^{1-\alpha } \end{array}$$

(A13) $$\begin{array}{cc} \; & \;=\sum_{x^{\prime }\in \mathrm{supp}\left(P_{X^{\prime }}\right)}P_{X^{\prime }}\left(x^{\prime }\right)\log\sum_{x\in \mathrm{supp}(P_{X})}B_{X|X^{\prime }}\left(x|x^{\prime }\right)\sum_{y}P_{Y|X}{\left(y|x\right)}^{\alpha }\;Q_{Y|X}{\left(y|x\right)}^{1-\alpha } \end{array}$$

(A14) $$\begin{array}{cc} \; & \;\geq \sum_{x^{\prime }\in \mathrm{supp}\left(P_{X^{\prime }}\right)}P_{X^{\prime }}\left(x^{\prime }\right)\sum_{x\in \mathrm{supp}(P_{X})}B_{X|X^{\prime }}\left(x|x^{\prime }\right)\log\sum_{y}P_{Y|X}{\left(y|x\right)}^{\alpha }\;Q_{Y|X}{\left(y|x\right)}^{1-\alpha } \end{array}$$

(A15) $$\begin{array}{cc} \; & \;=\sum_{x\in \mathrm{supp}(P_{X})}P_{X}\left(x\right)\left[\;\sum_{x^{\prime }\in \mathrm{supp}\left(P_{X^{\prime }}\right)}B_{X^{\prime }|X}\left(x^{\prime }|x\right)\right]\log\sum_{y}P_{Y|X}{\left(y|x\right)}^{\alpha }\;Q_{Y|X}{\left(y|x\right)}^{1-\alpha } \end{array}$$

(A16) $$\begin{array}{cc} \; & \;=\sum_{x\in \mathrm{supp}(P_{X})}P_{X}\left(x\right)\log\sum_{y}P_{Y|X}{\left(y|x\right)}^{\alpha }\;Q_{Y|X}{\left(y|x\right)}^{1-\alpha } \end{array}$$

(A17) $$\begin{array}{cc} \; & \;=\left(\alpha -1\right)\;D_{\alpha }^{c}(P_{Y|X}\|Q_{Y|X}\left|P_{X}\right), \end{array}$$

where (A10) follows from (30); (A11) follows from (41) and (42); (A12) follows from Hölder’s inequality; (A13) holds because $B_{X|X^{\prime }}\left(x|x^{\prime }\right)=0$ if $P_{X^{\prime }}\left(x^{\prime }\right)>0$ and $P_{X}\left(x\right)=0$; (A14) follows from Jensen’s inequality because log(·) is concave; (A15) follows from (40); (A16) holds because $P_{X}\left(x\right)>0$ and $P_{X^{\prime }}\left(x^{\prime }\right)=0$ imply $B_{X^{\prime }|X}\left(x^{\prime }|x\right)=0$, hence the expression in square brackets on the LHS of (A16) equals one; and (A17) follows from (30).

## Appendix D. Proof of Corollary 1

Applying Theorem 2 with $X^{\prime }≜\left\{1\right\}$ and the conditional PMF $B_{X^{\prime }|X}\left(x^{\prime }|x\right)≜1$, we obtain

(A18) $$\begin{array}{c} D_{\alpha }^{c}(P_{Y|X^{\prime }}\|Q_{Y|X^{\prime }}|P_{X^{\prime }})\leq D_{\alpha }^{c}(P_{Y|X}\|Q_{Y|X}\left|P_{X}\right). \end{array}$$

To complete the proof of (48), observe that

(A19) $$\begin{array}{cc} D_{\alpha }^{c}(P_{Y|X^{\prime }}\|Q_{Y|X^{\prime }}\left|P_{X^{\prime }}\right) & =D_{\alpha }^{c}(P_{Y}\|Q_{Y}\left|P_{X^{\prime }}\right) \end{array}$$

(A20) $$\begin{array}{cc} \; & =D_{\alpha }\left(P_{Y}\|Q_{Y}\right), \end{array}$$

where (A19) holds because (41) and (46) imply $P_{Y|X^{\prime }}\left(y|x^{\prime }\right)=P_{Y}\left(y\right)$ and because (42) and (47) imply $Q_{Y|X^{\prime }}\left(y|x^{\prime }\right)=Q_{Y}\left(y\right)$; and (A20) follows from Remark 1.

## Appendix E. Proof of Example 1

If α=∞, then it can be verified numerically that (53) holds for ϵ=0.1. Fix now α∈(1,∞). Then, for all ϵ∈(0,1),

(A21) $$\begin{array}{cc} D_{\alpha }\left(P_{Y}\|Q_{Y}^{\left(\epsilon \right)}\right) & =\frac{1}{\alpha -1}\log\left[0.5^{\alpha }\;{\left(1-\epsilon \right)}^{1-\alpha }+0.5^{\alpha }\;\epsilon^{1-\alpha }\right] \end{array}$$

(A22) $$\begin{array}{cc} \; & \geq \frac{1}{\alpha -1}\log\left[0.5^{\alpha }\;\epsilon^{1-\alpha }\right] \end{array}$$

(A23) $$\begin{array}{cc} \; & =\frac{\alpha }{\alpha -1}\log0.5+\log\frac{1}{\epsilon }. \end{array}$$

The RHS of (53) satisfies, for sufficiently small ϵ,

(A24) $$\begin{array}{cc} D_{\alpha }^{c}\left(P_{Y|X}^{\left(\epsilon \right)}\left\|Q_{Y}^{\left(\epsilon \right)}\right|P_{X}\right) & =0.5\cdot 0+0.5\cdot D_{\alpha }\left(P_{Y|X=1}^{\left(\epsilon \right)}\|Q_{Y}^{\left(\epsilon \right)}\right) \end{array}$$

(A25) $$\begin{array}{cc} \; & =\frac{0.5}{\alpha -1}\log\left[\epsilon^{\alpha }\;{\left(1-\epsilon \right)}^{1-\alpha }+{\left(1-\epsilon \right)}^{\alpha }\;\epsilon^{1-\alpha }\right] \end{array}$$

(A26) $$\begin{array}{cc} \; & =\frac{0.5}{\alpha -1}\log\left[\epsilon^{1-\alpha }\;\left({\left(1-\epsilon \right)}^{\alpha }+\epsilon^{2\alpha -1}\;{\left(1-\epsilon \right)}^{1-\alpha }\right)\right] \end{array}$$

(A27) $$\begin{array}{cc} \; & \leq \frac{0.5}{\alpha -1}\log\left[2\;\epsilon^{1-\alpha }\right] \end{array}$$

(A28) $$\begin{array}{cc} \; & =\frac{0.5}{\alpha -1}\log2+0.5\log\frac{1}{\epsilon }, \end{array}$$

where (A27) holds for sufficiently small ϵ because $\lim_{\epsilon ↓0}\left({\left(1-\epsilon \right)}^{\alpha }+\epsilon^{2\alpha -1}\;{\left(1-\epsilon \right)}^{1-\alpha }\right)=1$. Because $\lim_{\epsilon ↓0}\log\frac{1}{\epsilon }=\infty$, (53) follows from (A23) and (A28) for sufficiently small ϵ.

## Appendix F. Proof of Theorem 3

Observe that, for all $x^{\prime }\in X$ and all $y^{\prime }\in Y^{\prime }$,

(A29) $$\begin{array}{cc} P_{X}\left(x^{\prime }\right)\;P_{Y^{\prime }|X}\left(y^{\prime }|x^{\prime }\right) & =\sum_{x,\;y}P_{X}\left(x\right)\;P_{Y|X}\left(y|x\right)\;𝟙\left\{x^{\prime }=x\right\}\;A_{Y^{\prime }|XY}\left(y^{\prime }|x,y\right), \end{array}$$

(A30) $$\begin{array}{cc} P_{X}\left(x^{\prime }\right)\;Q_{Y^{\prime }|X}\left(y^{\prime }|x^{\prime }\right) & =\sum_{x,\;y}P_{X}\left(x\right)\;Q_{Y|X}\left(y|x\right)\;𝟙\left\{x^{\prime }=x\right\}\;A_{Y^{\prime }|XY}\left(y^{\prime }|x,y\right). \end{array}$$

Hence, (68) follows from (54) and

(A31) $$\begin{array}{c} D_{\alpha }\left(P_{X}P_{Y^{\prime }|X}\|P_{X}Q_{Y^{\prime }|X}\right)\leq D_{\alpha }\left(P_{X}P_{Y|X}\|P_{X}Q_{Y|X}\right), \end{array}$$

which follows from the data-processing inequality for the Rényi divergence by substituting ${𝟙}_{X^{\prime }=X}A_{Y^{\prime }|XY}$ for $A_{X^{\prime }Y^{\prime }|XY}$ in Proposition 1 (h).

## Appendix G. Proof of Theorem 4

Observe that, for all $x^{\prime }\in X^{\prime }$ and all $y^{\prime }\in Y$,

(A32) $$\begin{array}{cc} P_{X^{\prime }}\left(x^{\prime }\right)\;P_{Y|X^{\prime }}\left(y^{\prime }|x^{\prime }\right) & =\sum_{x,\;y}P_{X}\left(x\right)\;P_{Y|X}\left(y|x\right)\;B_{X^{\prime }|X}\left(x^{\prime }|x\right)\;𝟙\left\{y^{\prime }=y\right\}, \end{array}$$

(A33) $$\begin{array}{cc} P_{X^{\prime }}\left(x^{\prime }\right)\;Q_{Y|X^{\prime }}\left(y^{\prime }|x^{\prime }\right) & =\sum_{x,\;y}P_{X}\left(x\right)\;Q_{Y|X}\left(y|x\right)\;B_{X^{\prime }|X}\left(x^{\prime }|x\right)\;𝟙\left\{y^{\prime }=y\right\}. \end{array}$$

Hence, (73) follows from (54) and

(A34) $$\begin{array}{c} D_{\alpha }\left(P_{X^{\prime }}P_{Y|X^{\prime }}\|P_{X^{\prime }}Q_{Y|X^{\prime }}\right)\leq D_{\alpha }\left(P_{X}P_{Y|X}\|P_{X}Q_{Y|X}\right), \end{array}$$

which follows from the data-processing inequality for the Rényi divergence by substituting $B_{X^{\prime }|X}{𝟙}_{Y^{\prime }=Y}$ for $A_{X^{\prime }Y^{\prime }|XY}$ in Proposition 1 (h).
